# Budesonide-Formoterol Metered-Dose Inhaler vs Fluticasone-Salmeterol Dry-Powder Inhaler

**DOI:** 10.1001/jamainternmed.2025.2299

**Published:** 2025-07-07

**Authors:** Alexander S. Rabin, Sarah M. Seelye, Julien B. Weinstein, Cainnear K. Hogan, Taylor N. Whittington, Jennifer Cano, Shelie A. Miller, Catherine Kelley, Hallie C. Prescott

**Affiliations:** 1 Pulmonary Section, Veterans Affairs Ann Arbor Healthcare System, Ann Arbor, Michigan; 2 Department of Internal Medicine, University of Michigan, Ann Arbor; 3 Veterans Affairs Center for Clinical Management Research, Ann Arbor, Michigan; 4 School for Environment and Sustainability, University of Michigan, Ann Arbor; 5 Veterans Affairs Pharmacy Benefits Management Services, Hines, Illinois

## Abstract

**Importance:**

Transitioning from metered-dose inhalers to propellant-free dry-powder inhalers could reduce health care–related greenhouse gas emissions, but the clinical difference in outcomes that may be associated with this switch is uncertain.

**Objective:**

To evaluate the clinical difference in outcomes associated with a July 2021 Veterans Health Administration (VHA) formulary change that replaced budesonide-formoterol metered-dose inhaler with fluticasone-salmeterol dry-powder inhaler for the treatment of chronic obstructive pulmonary disease and asthma.

**Design, Setting, and Participants:**

This within-person, self-controlled case series (SCCS) and matched observational cohort study (cohort study) used data from the US Veterans Affairs health care system from January 2018 through December 2022. Veterans who were prescribed a combination inhaler before and after the formulary change were included in both the SCCS and cohort study. Data were analyzed between April 19, 2024, and April 4, 2025.

**Exposures:**

Treatment with budesonide-formoterol metered-dose inhaler vs fluticasone-salmeterol dry-powder inhaler.

**Main Outcomes and Measures:**

Rescue medication use (albuterol and prednisone fills), emergency department visits, and hospitalizations (all-cause, respiratory-related, and pneumonia-specific) were assessed.

**Results:**

Following the VHA formulary change, 260 268 patients switched from budesonide-formoterol metered-dose therapy to fluticasone-salmeterol dry-powder therapy. In the SCCS (median [IQR] age, 71 [62-75] years; 91% male), among patients who switched inhalers and experienced the adverse outcomes of interest, treatment with fluticasone-salmeterol dry-powder inhaler therapy was associated with a 10% decrease in albuterol fills (incidence rate ratio [IRR], 0.90 [95% CI, 0.90-0.91]), a 2% increase in prednisone fills (IRR, 1.02 [95% CI, 1.01-1.03]), a 5% increase in all-cause emergency department visits (IRR, 1.05 [95% CI, 1.04-1.06]), an 8% increase in all-cause hospitalizations (IRR, 1.08 [95% CI, 1.06-1.09]), a 10% increase in respiratory-related hospitalizations (IRR, 1.10 [95% CI, 1.07-1.14]), and a 24% increase in pneumonia-specific hospitalizations (IRR, 1.24 [95% CI, 1.17-1.31]). In the cohort study of 258 557 patients (mean [SD] age, 68.9 [11.3] years; 94% male), those who switched to a fluticasone-salmeterol dry-powder inhaler had no difference in mortality (1.89% vs 1.90%; adjusted absolute difference, −0.01 percentage points [95% CI, −0.12 to 0.10 percentage points]) but had increases in all-cause hospitalizations (16.14% vs 15.64%; adjusted absolute difference, 0.49 percentage points [95% CI, 0.21-0.78 percentage points]), respiratory-related hospitalizations (3.15% vs 2.74%; adjusted absolute difference, 0.41 percentage points [95% CI, 0.27-0.55 percentage points]), and pneumonia-related hospitalizations (1.15% vs 1.03%; adjusted absolute difference, 0.12 percentage points [95% CI, 0.04-0.21 percentage points]) at 180 days after the switch compared with matched patients who did not switch.

**Conclusions and Relevance:**

The study found that the VHA formulary transition from budesonide-formoterol metered-dose inhaler to fluticasone-salmeterol dry-powder inhaler was associated with increased health care utilization, suggesting potential harm and the need to reevaluate this policy change.

## Key Points

**Question:**

Is there a clinical difference in outcomes associated with transitioning from budesonide-formoterol metered-dose inhaler therapy to fluticasone-salmeterol dry-powder inhaler therapy for the treatment of chronic obstructive pulmonary disease and asthma?

**Findings:**

In this primary self-controlled case series and secondary matched cohort study of 260 268 and 258 577 US veterans, respectively, transition to fluticasone-salmeterol dry-powder inhaler therapy under a national formulary change was associated with higher rates of prednisone use and increased all-cause and respiratory-related emergency department visits and hospitalizations.

**Meaning:**

These results suggest that there was an increase in adverse health outcomes after the shift from budesonide-formoterol metered-dose inhaler therapy to fluticasone-salmeterol dry-powder inhaler therapy in the Veterans Health Administration.

## Teaser

This cohort study assesses the clinical difference in outcomes associated with a Veterans Health Administration formulary change that replaced budesonide-formoterol metered-dose inhaler therapy with fluticasone-salmeterol dry-powder inhaler therapy for the treatment of chronic obstructive pulmonary disease and asthma among veterans.

## Introduction

Metered-dose inhalers contain hydrofluorocarbon propellants—synthetic gases that trap heat in the atmosphere thousands of times more powerfully than carbon dioxide.^1,2,3^ To mitigate health care–related greenhouse gas emissions, several organizations, including England’s National Health Service,^4^ the Canadian Thoracic Society,^5^ and the National Asthma Council Australia,^6^ have recommended switching patients from metered-dose to propellant-free dry-powder inhalers when appropriate.

However, transitioning inhaler devices presents clinical challenges. Inhalers within the same therapeutic class (eg, inhaled corticosteroid plus long-acting β-agonist [LABA]) may vary in both medication composition and device characteristics. Metered-dose inhalers deliver medication to the lungs independent of inspiratory effort but require patients to coordinate actuation with inhalation, whereas dry-powder inhalers rely on rapid, deep inhalation to minimize oropharyngeal deposition, which may be difficult for patients with diminished lung function.^7^ Moreover, not all medications are available in both metered-dose inhaler and dry-powder inhaler formulations in the US, complicating a large-scale device transition.^8^

In July 2021, following a cost-based competitive bidding process, the Veterans Health Administration (VHA) replaced metered-dose budesonide-formoterol (Symbicort) with dry-powder fluticasone-salmeterol (Wixela Inhub) as the preferred inhaled corticosteroid plus LABA combination for patients with chronic obstructive pulmonary disease (COPD) and asthma.^9,10^ Prior studies comparing these medication combinations have yielded mixed results, with few accounting for differences in delivery device.^11,12,13,14^ In asthma, budesonide-formoterol has been associated with fewer exacerbations and improved symptom control compared with fluticasone-salmeterol.^15^ Additionally, the use of inhaled corticosteroid plus formoterol combinations in a single maintenance-and-reliever strategy has become a favored approach for asthma treatment.^16,17^ In contrast, a recent study comparing a fluticasone-containing, triple-combination dry-powder inhaler with a budesonide-containing, triple-combination metered-dose inhaler among patients with COPD found a modestly higher incidence of moderate to severe COPD exacerbation with the budesonide combination, while other outcomes were comparable.^18^

The VHA formulary change created a natural experiment to evaluate the clinical difference in outcomes associated with switching both medication (budesonide-formoterol vs fluticasone-salmeterol) and delivery device (metered-dose inhaler vs dry-powder inhaler) in a nationwide cohort of veterans. We hypothesized that clinical outcomes would be similar between budesonide-formoterol metered-dose and fluticasone-salmeterol dry-powder inhaler therapy, and that clinical equivalence could provide compelling evidence to support broader adoption of the less expensive^19^ and environmentally sustainable dry-powder alternative. To test this hypothesis, we conducted a primary within-person analysis and a secondary matched cohort analysis to assess the association between the formulary change and clinical outcomes.

## Methods

### Study Overview

We included all veterans from January 1, 2018, to December 31, 2022, who were prescribed a combination controller inhaler both before and after the July 2021 VHA national formulary change in which budesonide-formoterol metered-dose inhalers were replaced by fluticasone-salmeterol dry-powder inhalers as the preferred inhaled corticosteroid plus LABA combination for treatment of patients with COPD and asthma. The inhaler formulary change was approved by the Veterans Affairs (VA) Pharmacy Benefits Management services and National Formulary Committee following a competitively bid contract negotiation.^20^ The study was approved by the VA Ann Arbor, Michigan, institutional review board and deemed exempt from the need for consent under 45CFR§46, category 4 (secondary use of identifiable data). The study follows the Strengthening the Reporting of Observational Studies in Epidemiology (STROBE) reporting guideline.

### Data Collection and Definitions

Data were extracted from the VA Corporate Data Warehouse and linked to Medicare claims as part of the VA Study of a Real-World Inhaler Delivery Device Transition on Climate and Health Outcomes (VA-SWITCH).^21^ Demographic and clinical data included age, sex, COPD diagnosis, asthma diagnosis, smoking status, combat veteran status, and location by census region. Combination controller inhalers were defined as those containing 2 or more of the following medication classes: inhaled corticosteroid, LABA, and long-acting muscarinic antagonist from the VA national formulary anti-asthma therapeutic class,^9^ as described in the eMethods in Supplement 1. Respiratory diagnoses were determined using *International Statistical Classification of Diseases and Related Health Problems, Tenth Revision (ICD-10)* codes (COPD: J41*, J42*, J43*, J44*; asthma: J45*, J46*; and pneumonia: J09.X1, J10*-J18*, A01.03, A02.22, A37.01, A37.11, A37.81, A37.91, A54.84, B01.2, B05.2, B06.81, B77.81, J85.1, J22*). Measures of pulmonary function were extracted using previously described methods.^22^ Prescriptions for nebulized albuterol were standardized to inhaler equivalents; prednisone use was defined as the discrete number of prescriptions filled. As many veterans receive acute care outside the VA health system (eg, during an acute exacerbation of COPD),^23^ we extracted emergency department (ED) visits and hospitalizations from the VA health record, fee-based claims for VA-paid care in the community, and Medicare claims. Respiratory-related health care utilization for asthma, COPD, and pneumonia was determined by principal *ICD-10* code.

### Statistical Analysis

**Figure 1.**
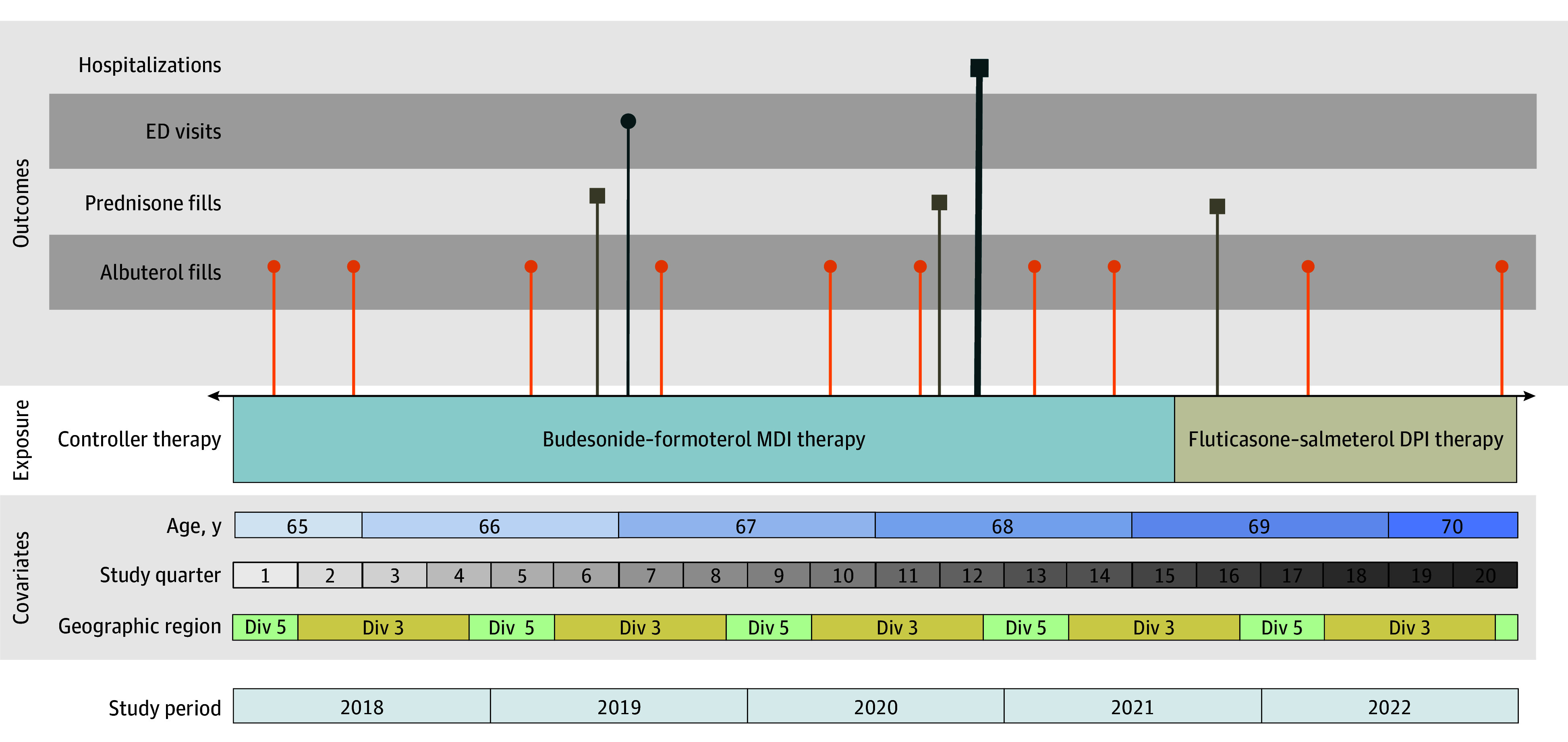
Schematic of the Self-Controlled Case Series Study Design The figure shows the timeline of exposures, outcomes, and time-varying covariates for a hypothetical patient who switches from budesonide-formoterol metered-dose inhaler (MDI) therapy to fluticasone-salmeterol dry-powder inhaler (DPI) therapy following the national formulary change. The patient is aged 65 to 70 years during the course of the study period and splits time between Michigan (census division 3 [div 3]) and Florida (census division 5 [div 5]). Using the self-controlled case-series method, outcome rates are compared across exposure periods, adjusting for the time-varying covariates of age, study quarter, and geographic region. eFigure 4 in Supplement 1 demonstrates how this hypothetical patient timeline is represented in the study dataset. ED indicates emergency department.

We conducted a primary self-controlled case series (SCCS) analysis ^24,25,26^ and a secondary matched observational cohort study (cohort study). An SCCS is a within-person analysis in which patients serve as their own controls. Using this analytic approach, we determined the relative incidence (incidence rate ratio [IRR]) of adverse clinical outcomes when receiving treatment with budesonide-formoterol metered-dose inhaler vs fluticasone-salmeterol dry-powder inhaler therapy. By definition, the IRR can be calculated only among patients who ever experience the outcome of interest, so the relative incidence cannot be applied to the full study cohort. As a within-person analysis, an SCCS inherently adjusts for time-invariant characteristics (eg, sex) and avoids confounding from between-individual differences. We additionally adjusted for the following time-varying covariates: age, calendar quarter (to account for seasonality), geographic region, and interaction between quarter and region (to account for geographic variation in respiratory viral illness) as displayed in Figure 1.

We estimated IRRs using conditional fixed-effects Poisson regression by comparing the incidence of adverse health outcomes between exposure periods. Budesonide-formoterol metered-dose inhaler and fluticasone-salmeterol dry-powder inhaler exposure periods were determined using longitudinal prescription fill data. Because inhaler use can only be inferred from these data and not measured directly, we considered 3 approaches to calculating inhaler exposure periods: no grace period (assumes inhaler is used as prescribed; a 30-day inhaler lasts 30 days); 33% grace period (assumes inhaler is used 75% of the time; a 30-day inhaler lasts 40 days); and 100% grace period (assumes inhaler is used 50% of the time; a 30-day inhaler lasts 60 days), as explained in eFigure 1 in Supplement 1. The 33% grace period approach was used in the primary analysis as it reflects general patient adherence.^27,28^ We additionally completed analyses excluding patients with longer than a 3-month gap between inhaler exposure periods.

We considered 8 outcomes: albuterol inhaler-equivalent fills; prednisone fills; all-cause, respiratory-related, and pneumonia-specific ED visits; and all-cause, respiratory-related, and pneumonia-specific hospitalizations. We completed subgroup analyses for each outcome by sex, respiratory diagnosis (COPD and asthma), smoking status (current, former, and never), combat veteran status, preswitch rescue medication use, preswitch ED utilization, and preswitch hospitalization. We completed sensitivity analyses in which pneumonia ED visits occurring shortly prior to pneumonia hospitalization were excluded and in which results were stratified by patients experiencing 1 vs more events of interest during the study period. Finally, we completed exploratory subgroup analyses by forced expiratory volume in the first second (FEV_1_) percent predicted in a subset of patients with lung function measurement.

Because an SCCS analysis may have temporal confounding and cannot generate absolute risk differences, we additionally completed a cohort study. Full design features of this secondary analysis are presented in eTable 1 in Supplement 1. Briefly, we compared outcomes of patients who switched to fluticasone-salmeterol dry-powder inhaler therapy vs continued receiving non–fluticasone-salmeterol inhaler therapy. Clinical outcomes were compared at 90 and 180 days after enrollment, defined as the date of first fluticasone-salmeterol dry-powder inhaler fill after the national formulary change for the switched group or first non–fluticasone-salmeterol inhaler fill in the not switched group. To control for confounding, we used both weighting and regression adjustment to balance switched vs not switched groups. First, using coarsened exact matching weights,^29^ we balanced the populations on the following characteristics measured at enrollment: age, sex, COPD diagnosis, asthma diagnosis, ED utilization in prior year, hospitalization in prior year, albuterol fills in prior year, prednisone use in prior year, smoking status, and geographic region. We assessed covariate balance using standardized mean differences. Second, using regression in the weighted populations, we adjusted for the same covariates to further control for confounding and yield precise estimates associated with the treatment.^30^

Finally, we estimated the difference in outcomes of the formulary change associated with the inhaler-related greenhouse gas emissions, as described in the eMethods in Supplement 1. Analyses were performed from April 19, 2024, to April 4, 2025, using Stata MP, version 18.0 (StataCorp LLC). The statistical code is available at GitHub.^31^ Statistical significance was defined as a 95% CI excluding 1.

## Results

### Demographics and Device Switching Patterns

During the study period, 347 486 patients were prescribed combination inhaler therapy both before and after the formulary change. Of these patients, 260 268 met SCCS inclusion criteria: prescription of budesonide-formoterol metered-dose inhaler therapy before the formulary change followed by prescription of fluticasone-salmeterol dry-powder inhaler therapy after the formulary change (eFigure 2 in Supplement 1).

**Figure 2.**
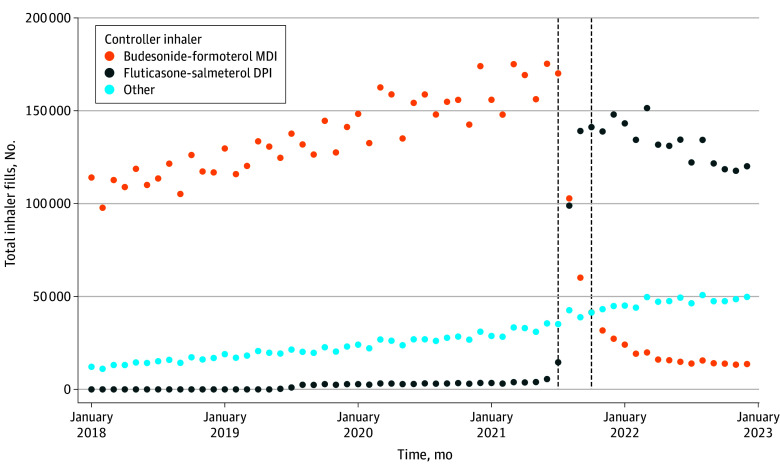
Inhaler Dispensing Before and After the National Formulary Change in the Self-Controlled Case Series Cohort The figure shows the number of monthly controller inhalers dispensed (budesonide-formoterol metered-dose inhaler [MDI], fluticasone-salmeterol dry-powder inhaler [DPI]) or other combination inhaled corticosteroid, long-acting β-agonist, or long-acting muscarinic antagonist from January 1, 2018, to December 31, 2022, among 347 486 patients who received controller inhalers during the study period both before and after the formulary switch. The increasing number of budesonide-formoterol MDIs over time reflects the larger number of patients included in the cohort leading up to the formulary change. There were 272 538 patients prescribed controller inhalers prior to the formulary switch who were excluded from the study cohort because they were not dispensed a controller inhaler after the formulary switch. The vertical dashed lines indicate the formulary change period from July 1, 2021, to September 30, 2021.

The SCCS cohort had a median (IQR) age of 71 (62-75) years; 9% were female and 91% were male. Most patients had COPD (69%), 32% had asthma, and 81% had a history of smoking. In the year preceding the formulary change, 82% filled an albuterol prescription, 16% filled a prednisone prescription, 29% had an ED visit, and 24% were hospitalized (Table 1). As expected, after the formulary change, prescription fills of budesonide-formoterol metered-dose inhalers decreased markedly, while prescription fills for fluticasone-salmeterol dry-powder inhalers increased (Figure 2). Total exposure time on budesonide-formoterol metered-dose inhaler therapy and fluticasone-salmeterol dry-powder inhaler therapy was 485 695 and 174 814 person-years, respectively (eTable 2 in Supplement 1).

**Table 1.** Baseline Characteristics of Self-Controlled Case Series Cohort That Switched From Budesonide-Formoterol Metered-Dose Inhaler to Fluticasone-Salmeterol Dry-Powder Inhaler Therapy^a^

Characteristic	Participants, No. (%)^b^
No. of patients	260 268
Age, median (IQR), y^c^	71 (62-75)
Sex	
Female	22 354 (8.6)
Male	237 914 (91.4)
Respiratory diagnosis^c^^,^^d^	
Chronic obstructive pulmonary disease	180 013 (69.2)
Asthma	84 300 (32.4)
Smoking status^c^	
Current	72 389 (27.8)
Former	136 944 (52.6)
Never	50 935 (19.6)
Combat veteran status^e^	79 196 (30.4)
Geographical census region^c^	
South	116 494 (44.8)
Midwest	62 935 (24.2)
West	48 317 (18.6)
Northeast	32 522 (12.5)
Medication and health care utilization in the year prior to inhaler switch	
Filled albuterol	213 648 (82.1)
Filled prednisone	40 588 (15.6)
Had an emergency department visit	76 210 (29.3)
Had a hospitalization	61 318 (23.6)

^a^ Additional data sources and methods are provided in the eMethods in Supplement 1.

^b^ Percentages may not total 100 because of rounding.

^c^ At time of inhaler switch.

^d^ Respiratory diagnosis determined using *International Statistical Classification of Diseases and Related Health Problems, Tenth Revision* codes. Patients may have more than 1 respiratory diagnosis assigned.

^e^ Combat veteran status was obtained from the electronic health record and the US Veterans Eligibility Trends and Statistics data source.

### Primary Self-Controlled Case Series Analysis

**Figure 3.**
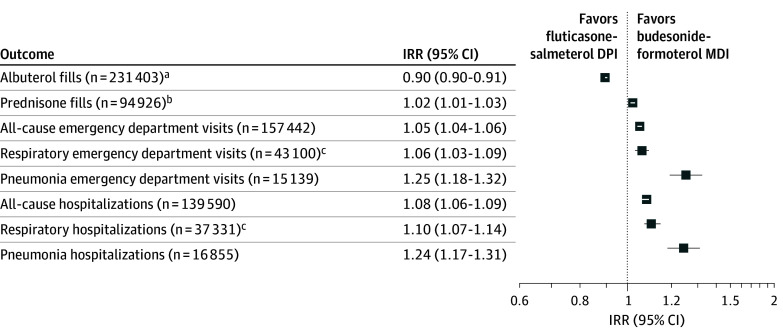
Clinical Outcomes Among Patients Who Switched Inhalers in the Self-Controlled Case Series Cohort The figure shows the relative incidence of adverse health outcomes during periods of fluticasone-salmeterol dry-powder inhaler (DPI) use vs periods of budesonide-formoterol metered-dose inhaler (MDI) use among those who experienced the outcomes of interest. In this analysis, we allowed a 33% grace period for inhaler fills to define exposure periods. IRR indicates incidence rate ratio. ^a^Albuterol represented as inhaler-equivalent medication fills. ^b^Prednisone represented as discrete courses of the medication filled. ^c^Respiratory cause defined as a principal diagnosis code of chronic obstructive pulmonary disease or asthma.

Among patients ever experiencing the respective outcomes of interest, fluticasone-salmeterol dry-powder inhaler periods were associated with a 10% reduction in albuterol fills (IRR, 0.90; [95% CI, 0.90-0.91]) compared with periods of budesonide-formoterol metered-dose inhaler therapy. However, fluticasone-salmeterol dry-powder inhaler periods were associated with a 2% increase in prednisone fills (IRR, 1.02 [95% CI, 1.01-1.03]), a 5% increase in all-cause ED visits (IRR, 1.05 [95% CI, 1.04-1.06]), a 6% increase in respiratory-related ED visits (IRR, 1.06 [95% CI, 1.03-1.09]), a 25% increase in pneumonia-specific ED visits (IRR, 1.25 [95% CI, 1.18-1.32]), an 8% increase in all-cause hospitalizations (IRR, 1.08 [95% CI, 1.06-1.09]), a 10% increase in respiratory-related hospitalizations (IRR, 1.10 [95% CI, 1.07-1.14]), and a 24% increase in pneumonia-specific hospitalizations (IRR, 1.24 [95% CI, 1.17-1.31]) compared with periods of budesonide-formoterol metered-dose inhaler therapy (Figure 3). Thus, among 16 855 patients hospitalized for pneumonia during the study period, the incidence of pneumonia hospitalization was 24% higher during periods on fluticasone-salmeterol compared with periods on budesonide-formoterol.

### Sensitivity, Subgroup, and Exploratory Analyses

All results of the primary analysis remained significant when corrected for multiple comparisons using the Hochberg procedure. Findings were consistent across sensitivity analyses using different approaches to defining inhaler exposure periods: excluding patients with gaps in treatment, excluding pneumonia ED visits near to pneumonia hospitalizations, and stratifying by patients experiencing 1 vs more events of interest (eTables 3-5 in Supplement 1). Sensitivity analyses considering different grace periods and exclusion of patients with longer than a 3-month gap in controller inhaler use are shown in eTable 3 in Supplement 1. The results were also consistent across subgroups defined by sex, respiratory diagnosis, smoking status, combat veteran status, preswitch rescue medication use, and preswitch health care utilization (eFigure 3 in Supplement 1). Fluticasone-salmeterol dry-powder inhaler periods were associated with decreased albuterol fills compared with budesonide-formoterol metered-dose inhaler periods across subgroups. However, fluticasone-salmeterol dry-powder inhaler periods were largely associated with an increased incidence of other adverse outcomes across subgroups, including among patients with either COPD or asthma. Findings were also consistent in exploratory subgroup analyses by FEV_1_ percent predicted (eTable 6 and eTable 7 in Supplement 1). Estimated inhaler-related greenhouse gas emissions decreased following the formulary change, with mean emissions per veteran in the SCCS cohort of 279.8 kg of carbon dioxide equivalents in 2018 vs 101.0 kg of carbon dioxide equivalents in 2022 (eTable 8 in Supplement 1).

### Secondary Matched Observational Cohort Study

In the cohort study, outcomes of 167 331 patients who switched to fluticasone-salmeterol dry-powder inhaler therapy (mean [SD] age, 68.9 [11.5] years; 6% female and 94% male) were compared with outcomes of 91 226 patients (mean [SD] age, 68.9 [11.0] years; 6% female and 94% male) who continued receiving non–fluticasone-salmeterol inhaler therapy. Switched and not switched groups were well balanced on baseline characteristics after weighting (eTable 9 in Supplement 1). Patients who switched to fluticasone-salmeterol dry-powder inhaler therapy vs continued receiving a non–fluticasone-salmeterol inhaler had no significant difference in mortality at 90 days (0.46% vs 0.52%; adjusted absolute difference, −0.05 percentage points [95% CI, −0.11 to 0.00 percentage points]) or 180 days (1.89% vs 1.90%; adjusted absolute difference, −0.01 percentage points [95% CI, −0.12 to 0.10 percentage points]). In contrast to the SCCS, there was no change in albuterol fills (adjusted absolute difference, 0.05 fills [95% CI, −0.03 to 0.13]) or prednisone fills (adjusted absolute difference, 0.002 fills [95% CI, −0.004 to 0.008]) at 180 days. However, switched patients had increased all-cause hospitalizations (16.14% vs 15.64%; adjusted absolute difference, 0.49 percentage points [95% CI, 0.21-0.78 percentage points]), respiratory-related hospitalizations (3.15% vs 2.74%, adjusted absolute difference, 0.41 percentage points [95% CI, 0.27-0.55 percentage points]), and pneumonia-related hospitalizations (1.15% vs 1.03%; adjusted absolute difference, 0.12 percentage points [95% CI, 0.04-0.21 percentage points]) at 180 days (Table 2). Health outcomes were also consistent at 90 days (eTable 10 in Supplement 1) and in analyses using weighting without covariate adjustment (eTable 11 and eTable 12 in Supplement 1).

**Table 2.** Estimated 180-Day Health Outcomes (Adjusted) of Switched vs Not Switched Patients in a Matched Observational Cohort Study^a^

Outcome^b^	Adjusted %		Adjusted odds ratio (95% CI)	Adjusted absolute difference, % (95% CI)
	Switched (n = 167 331)	Not switched (n = 91 226)		
Albuterol fills^c^	3.47^d^	3.43^d^	1.01 (0.99 to 1.04)^e^	0.05 (−0.03 to 0.13)
Prednisone fills^f^	0.21^d^	0.21^d^	1.01 (0.98 to 1.04)^e^	0.002 (−0.004 to 0.008)
All-cause ED visits	19.45	18.31	1.09 (1.07 to 1.12)	1.14 (0.84 to 1.45)
Respiratory ED visits	3.29	2.97	1.12 (1.06 to 1.17)	0.32 (0.18 to 0.46)
Pneumonia ED visits	0.96	0.83	1.15 (1.05 to 1.26)	0.12 (0.04 to 0.20)
All-cause hospitalizations	16.14	15.64	1.04 (1.02 to 1.07)	0.49 (0.21 to 0.78)
Respiratory hospitalizations	3.15	2.74	1.16 (1.10 to 1.22)	0.41 (0.27 to 0.55)
Pneumonia hospitalizations	1.15	1.03	1.12 (1.04 to 1.22)	0.12 (0.04 to 0.21)
Mortality	1.89	1.90	0.99 (0.94 to 1.06)	−0.01 (−0.12 to 0.10)

Abbreviation: ED, emergency department.

^a^ Patients who switched were transitioned from a budesonide-formoterol metered-dose inhaler to a fluticasone-salmeterol dry-powder inhaler. Patients who were not switched continued receiving a non–fluticasone-salmeterol dry-powder inhaler. Among patients switched vs not switched, there was no difference in mortality and rescue medication use; there were small increases in all-cause and respiratory-related health care utilization.

^b^ Outcomes were estimated using logistic or Poisson regression, and predictive margins are reported for each treatment group.

^c^ Albuterol represented as inhaler-equivalent medication fills.

^d^ Adjusted count.

^e^ Incidence rate ratio (95% CI).

^f^ Prednisone represented as discrete courses of the medication filled.

## Discussion

The VHA national formulary change from a metered-dose inhaler (budesonide-formoterol) to a dry-powder inhaler (fluticasone-salmeterol) was associated with increased prednisone use and higher health care utilization. The findings of this study were robust across several sensitivity analyses, subgroup analyses, and 2 contrasting analytical approaches.

A key question raised by our study is whether the increased incidence of adverse outcomes among patients who switched to the dry-powder fluticasone-salmeterol inhaler was associated with the medication, the device, or other factors. The answer likely involves all 3 factors.

First, the pharmacological properties of the inhaled corticosteroid and LABA components differ. Although industry-sponsored head-to-head comparisons of budesonide-formoterol vs fluticasone-salmeterol combinations^14^ have shown similar clinical efficacy, other studies indicate that fluticasone is associated with a higher pneumonia risk than budesonide,^13,32,33^ likely due to its more sustained systemic^34^ and local^35^ immunosuppressive effects. In the present SCCS, among 16 855 of 260 268 patients who experienced a pneumonia hospitalization during the study period, there was a 24% increase in the relative incidence of pneumonia hospitalization during periods of fluticasone-salmeterol use compared with budesonide-formoterol use. In the secondary cohort study, patients who switched to fluticasone-salmeterol had a 0.12% increase (1.15% vs 1.03%) in pneumonia hospitalization risk in the 180 days following the formulary change, translating to approximately 310 additional pneumonia hospitalizations if all 258 557 patients in the analysis switched inhalers (vs none being switched). By comparison, a study by Feldman et al^18^ found no increased pneumonia risk with the fluticasone-containing triple therapy in COPD, possibly due to the distinct chemical profile^36^ and longer duration of action of fluticasone furoate vs fluticasone propionate. Additionally, the switch to salmeterol, a LABA with a slower onset of action than formoterol,^37,38^ may have negatively affected patient perceptions of inhaler efficacy, particularly as guidelines increasingly favor formoterol-containing combinations for asthma.^16,39^

Second, differences in drug delivery mechanisms may have influenced outcomes. Although clinical trials in Europe have demonstrated comparable efficacy between metered-dose inhalers and dry-powder inhalers,^40,41^ dry-powder inhalers have a smaller market share in the US.^1,3^ Veterans’ relative unfamiliarity with dry-powder inhalers may have led to negative perceptions and decreased tolerance, worsening disease control. While our exploratory analysis did not reveal major differences in outcomes based on baseline disease severity (ie, mild to moderate vs severe to very severe obstructive lung physiology), older patients with COPD may also have struggled to generate sufficient inspiratory force for effective dry-powder drug delivery.

Third, the formulary change may have disrupted established treatment routines, with evidence suggesting that forced device switching can decrease medication adherence, lead to errors in inhalation technique, and worsen clinical outcomes.^42,43^ For example, a 2016 payer-initiated formulary change from a metered-dose inhaler to a dry-powder inhaler documented poorer lung function among those who switched devices.^44^ In comparison, a large SCCS analysis in the UK found no increase in health care utilization among patients who switched inhalers, but most participants were maintained on the same drug, and three-quarters did not switch device classes.^24^

Implementation strategies likely play a role in lessening the impact associated with inhaler formulary changes: how transitions are implemented may be as important as the choice of drug or device. Although the VHA formulary change was determined centrally following an evidence review, uptake of the policy varied across facilities,^10^ as did efforts to provide inhaler training—particularly during the COVID-19 pandemic when access to VHA specialty care was disrupted.^45^ Structured inhaler education and clear patient and health care professional communication during formulary changes are essential to minimize interruptions in care.^46^ Regular reassessment of inhalation technique and peak flow monitoring may also improve dry-powder drug delivery among patients who switch to this device class.^47^

Our results highlight the tension between reducing inhaler costs, improving clinical outcomes, and addressing the environmental impact of care delivery. While the formulary change likely lowered inhaler expenditures and reduced direct inhaler-related greenhouse gas emissions—the equivalent of taking approximately 6000 gasoline-powered passenger vehicles off the road for 1 year—higher health care utilization could offset these climate benefits by increasing resource-intensive ED visits and hospitalizations. For health systems aiming to align with decarbonization goals,^48^ interventions must optimize clinical outcomes and net greenhouse gas emissions while balancing costs.

### Strengths and Limitations

The strengths of this study include leveraging a national policy change to assess clinical outcomes, a large sample size, and a within-person design that accounts for time-invariant confounders. Consistency across exposure definitions and methodologies, including both an SCCS and a matched observational cohort analysis, further supports the validity of our findings.

Important limitations must also be considered for this study. First, the cohort included older male veterans with COPD, limiting generalizability to younger patients and patients with asthma. However, findings were consistent in subgroup analyses. Second, although we used longitudinal dispensing data, we could not measure inhaler use directly. However, findings were consistent across sensitivity analyses using different approaches to operationalizing inhaler exposure periods. While our primary SCCS analysis used a 33% grace period when defining inhaler exposure periods, which may introduce bias,^49^ it reflects general patient adherence and yielded consistent findings as alternate approaches. Third, while we adjusted for geographic region and study quarter in the SCCS and compared outcomes among patients treated contemporaneously in the cohort study, residual confounding from changes in care-seeking behaviors during the COVID-19 pandemic remains possible. Additionally, use of study quarter to account for seasonality may not fully capture seasonal variation in respiratory events. Fourth, we focused on short-term clinical outcomes, not longer-term impacts associated with COPD or asthma progression nor quality of life. Fifth, use of an SCCS to study recurrent events may result in bias. However, the results were consistent in sensitivity analyses stratified by the number of events. Sixth, exploratory subgroup analyses by FEV_1_ percent predicted could be done only in a smaller, nonrandom sample of veterans with available data. Seventh, the findings are specific to one formulary change and may not apply to other inhaler transitions. Additionally, while cost savings from the formulary change allowed the VA to reallocate funds to other health care services, we could not quantify these benefits to understand the holistic impact associated with the formulary change.

## Conclusions

This study found that the change from a budesonide-formoterol metered-dose inhaler to a fluticasone-salmeterol dry-powder inhaler for treatment of COPD and asthma was associated with increased health care utilization and potential clinical harm among veterans. Additional research is needed to refine inhaler switching strategies across large health systems to optimize patient outcomes and planetary health.

## Notes

Supplement 1.
eMethods.
**eTable 1.** Matched Observational Cohort Study Design **eTable 2.** Total Exposure Time and Outcomes During Exposure Periods **eTable 3.** Sensitivity Analysis of Fluticasone-salmeterol Dry-Powder Inhaler vs Budesonide-formoterol Metered-Dose Inhaler on Clinical Outcomes by Approach to Operationalizing Inhaler Exposure Periods **eTable 4.** Sensitivity Analysis Excluding Emergency Department Visits Occurring Near to Hospitalization for Pneumonia **eTable 5.** Sensitivity Analysis Examining Patients With 1 vs 2+ Events During the Study Period **eTable 6.** Ratio of Forced Expiratory Volume in 1 Second (FEV1) to Forced Vital Capacity and FEV1 Percent Predicted in the Study Cohort **eTable 7.** Impact of Fluticasone-salmeterol Dry-Powder Inhaler vs Budesonide-formoterol Metered-Dose Inhaler on Clinical Outcomes by Forced Expiratory Volume in 1 Second Percent Predicted **eTable 8.** Estimated Inhaler-Related Greenhouse-Gas Emissions **eTable 9.** Covariate Balance of Switched vs Not-Switched Populations in the Matched Observational Cohort Study **eTable 10.** Estimated 90-Day Health Outcomes (Adjusted) of Switched vs Not-Switched Patients in a Matched Observational Cohort Study **eTable 11.** Estimated 180-Day Health Outcomes (Unadjusted) of Switched vs Not-Switched Patients in the Matched Observational Cohort Study **eTable 12.** Estimated 90-Day Health Outcomes (Unadjusted) of Switched vs Not-Switched Patients in the Matched Observational Cohort Study **eFigure 1.** Three Approaches to Operationalizing Inhaler Exposure Periods: No Grace Period, 33% Grace Period, and 100% Grace Period **eFigure 2.** Patients Included in the Study Cohort **eFigure 3.** Clinical Outcomes Stratified by Subgroups **eFigure 4.** Self-Controlled Case Series Study Design, Expanded

Supplement 2.
Data Sharing Statement
