# Supplementary material for: Budesonide-Formoterol Metered-Dose Inhaler vs Fluticasone-Salmeterol Dry-Powder Inhaler
Source: JAMA Intern Med. 2025 Jul 7;185(8):1005–13. doi: 10.1001/jamainternmed.2025.2299 (PMC12235531; doi:10.1001/jamainternmed.2025.2299)
Supplement: Supplement 1. — eMethods. eTable 1. Matched Observational Cohort Study Design eTable 2. Total Exposure Time and Outcomes During Exposure Periods eTable 3. Sensitivity Analysis of Fluticasone-salmeterol Dry-Powder Inhaler vs Budesonide-formoterol Metered-Dose Inhaler on Clinical Outcomes by Approach to Operationalizing Inhaler Exposure Periods eTable 4. Sensitivity Analysis Excluding Emergency Department Visits Occurring Near to Hospitalization for Pneumonia eTable 5. Sensitivity Analysis Examining Patients With 1 vs 2+ Events During the Study Period eTable 6. Ratio of Forced Expiratory Volume in 1 Second (FEV1) to Forced Vital Capacity and FEV1 Percent Predicted in the Study Cohort eTable 7. Impact of Fluticasone-salmeterol Dry-Powder Inhaler vs Budesonide-formoterol Metered-Dose Inhaler on Clinical Outcomes by Forced Expiratory Volume in 1 Second Percent Predicted eTable 8. Estimated Inhaler-Related Greenhouse-Gas Emissions eTable 9. Covariate Balance of Switched vs Not-Switched Populations in the Matched Observational Cohort Study eTable 10. Estimated 90-Day Health Outcomes (Adjusted) of Switched vs Not-Switched Patients in a Matched Observational Cohort Study eTable 11. Estimated 180-Day Health Outcomes (Unadjusted) of Switched vs Not-Switched Patients in the Matched Observational Cohort Study eTable 12. Estimated 90-Day Health Outcomes (Unadjusted) of Switched vs Not-Switched Patients in the Matched Observational Cohort Study eFigure 1. Three Approaches to Operationalizing Inhaler Exposure Periods: No Grace Period, 33% Grace Period, and 100% Grace Period eFigure 2. Patients Included in the Study Cohort eFigure 3. Clinical Outcomes Stratified by Subgroups eFigure 4. Self-Controlled Case Series Study Design, Expanded [file jamainternmed-e252299-s001.pdf]

Supplementary Online Content

Rabin AS, Seelye SM, Weinstein JB, et al. Budesonide-formoterol metered-dose inhaler vs fluticasone-salmeterol dry-powder inhaler. Published online June 30, 2025. *JAMA Intern Med*. doi:10.1001/jamainternmed.2025.2299

eMethods.

eTable 1. Matched Observational Cohort Study Design

eTable 2. Total Exposure Time and Outcomes During Exposure Periods

eTable 3. Sensitivity Analysis of Fluticasone–Salmeterol Dry-Powder Inhaler vs Budesonide–Formoterol Metered-Dose Inhaler on Clinical Outcomes by Approach to Operationalizing Inhaler Exposure Periods

eTable 4. Sensitivity Analysis Excluding Emergency Department Visits Occurring Near to Hospitalization for Pneumonia

eTable 5. Sensitivity Analysis Examining Patients With 1 vs 2+ Events During the Study Period

eTable 6. Ratio of Forced Expiratory Volume in 1 Second (FEV1) to Forced Vital Capacity and FEV1 Percent Predicted in the Study Cohort

eTable 7. Impact of Fluticasone–Salmeterol Dry-Powder Inhaler vs Budesonide–Formoterol Metered-Dose Inhaler on Clinical Outcomes by Forced Expiratory Volume in 1 Second Percent Predicted

e**Table 8.** Estimated Inhaler-Related Greenhouse-Gas Emissions

eTable 9. Covariate Balance of Switched vs Not-Switched Populations in the Matched Observational Cohort Study

eTable 10. Estimated 90-Day Health Outcomes (Adjusted) of Switched vs Not-Switched Patients in a Matched Observational Cohort Study

eTable 11. Estimated 180-Day Health Outcomes (Unadjusted) of Switched vs Not-Switched Patients in the Matched Observational Cohort Study

eTable 12. Estimated 90-Day Health Outcomes (Unadjusted) of Switched vs Not-Switched Patients in the Matched Observational Cohort Study

© 2025 Rabin AS et al. *JAMA Intern Med.*

eFigure 1. Three Approaches to Operationalizing Inhaler Exposure Periods: No Grace Period, 33% Grace Period, and 100% Grace Period

eFigure 2. Patients Included in the Study Cohort

eFigure 3. Clinical Outcomes Stratified by Subgroups

eFigure 4. Self-Controlled Case Series Study Design, Expanded

eReferences.

This supplementary material has been provided by the authors to give readers additional information about their work.

© 2025 Rabin AS et al. *JAMA Intern Med.*

eMethods

Study Cohorts

We identified all patients who had outpatient medications filled in the Veterans Health Administration (VHA) between January 1, 2018, and September 30, 2021. Of these, we identified patients who received an RE109 combination inhaler[1] during two time periods: 1) January 1, 2018, through September 30, 2021, and 2) October 1, 2021, through December 31, 2022. Time period #1 included the "pre-switch" period that occurred prior to the Veterans Affairs (VA) formulary change on July 1, 2021, as well as a 3-month "washout" period from July 1, 2021, through September 30, 2021. Time period #2 included the "post-switch" period between October 1, 2021, and December 31, 2022. We then identified patients who received 1) a budesonide–formoterol metered-dose inhaler or other combination controller inhaler during the pre-switch or washout periods and 2) a fluticasone–salmeterol dry powder inhaler during the washout or post-switch periods. The other combination controller inhalers that were identified during the pre-switch or washout periods included mometasone–formoterol (Dulera HFA; Organon), budesonide–glycopyrrolate–formoterol (Breztri Aerosphere; AstraZeneca), fluticasone–vilanterol (Breo Ellipta; GlaxoSmithKline), umeclidinium–vilanterol (Anoro Ellipta; GlaxoSmithKline), fluticasone–salmeterol (Advair Diskus; GlaxoSmithKline), fluticasone–salmeterol (AirDuo RespiClick; Teva), fluticasone–salmeterol (Advair HFA; GlaxoSmithKline), fluticasone–umeclidinium–vilanterol (Trelegy Ellipta; GlaxoSmithKline), and tiotropium–olodaterol (Stiolto Respimat; Boehringer Ingelheim).

Outpatient medication data were extracted from the VA Corporate Data Warehouse (CDW) in August 2023. The CDW is a central repository of clinical and administrative data from the VHA that contains electronic health records for all patients across the national health system. Data on combination controller inhaler fills were extracted from CDW's outpatient pharmacy domain in the RxOutpatFill table using the date and time that medications were filled (ReleaseDateTime) and the amount of medication dispensed (QtyNumeric). When more than one type of combination controller inhaler was prescribed on the same day, we prioritized prescriptions in the following order: (1) fluticasone–salmeterol dry powder inhaler, (2) budesonide–formoterol metered-dose inhaler, and (3) other combination inhaler.

Addresses

The primary geographic unit that we used for patient residence was the United States census region (South, Midwest, West, and Northeast). We matched patient addresses to each census region within the US using patient zip codes available in the Outpat.Visit table in the Outpatient domain. Patient zip codes were mapped to states and to the census region and division associated with those states. Patient addresses that fell outside of a US census region were assigned international addresses with census regions that were geographically closest in location (eg, Puerto Rico was assigned the same census region as Florida), as shown below.

International Location	Census Region
Nova Scotia	Northeast (1)
New Brunswick	Northeast (1)
Ontario	Midwest (2)
Mexico	South (3)
Puerto Rico	South (3)
Virgin Islands	South (3)
American Samoa	West (4)
Armed Forces Pacific	West (4)
British Columbia	West (4)
Federated States of Micronesia	West (4)
Guam	West (4)
Northern Mariana Islands	West (4)
Palau	West (4)
Philippines	West (4)
US Minor Outlying Islands	West (4)
Yukon Territory	West (4)

Patients who entered the study period without an address on file at the time of their first combination controller inhaler prescription were assigned the earliest address that appeared in the study period. All patients without an address prior to their first address on record were backfilled using their initial address during the study period. We made this decision for

two reasons. First, only 2.9% of patients (N=10,100 of 347,486) entered the study period without an address, and second, among our cohort, very few patients moved to a different census region during the study period (the median number of census region changes during the study period was 0). In total, 797 patients (0.2%) had missing address information. For these patients, we used the region of the VA facility that prescribed the combination controller inhaler as a proxy for the patient's address region.

Smoking Status

We identified patients' smoking status on the day of their transition to a fluticasone–salmeterol dry powder inhaler using data in the health factor domain of CDW. Smoking status was categorized as current smoker, former smoker, and never smoker. If smoking status was not recorded on the day of a patient's inhaler switch, we used data from the most recent date within two years prior to their inhaler switch date to identify smoking status. If smoking status was still unavailable, we used the most recent date in the one year following their inhaler switch date to report smoking status.

For patients who did not transition to the fluticasone–salmeterol dry-powder inhaler and thus did not have a switch date (N=87,218), we used the VA national formulary date of July 1, 2021, to identify their smoking status. As we did for patients who transitioned to a fluticasone–salmeterol dry-powder inhaler, if smoking status was not available on July 1, 2021, we prioritized smoking status in the earliest date within two years prior to July 1, 2021, and if unavailable, one year following July 1, 2021.

Respiratory Diagnoses

Respiratory diagnoses, including asthma, chronic obstructive pulmonary disease (COPD), and pneumonia, were identified using *International Classification of Diseases, 10th Revision* (ICD-10) codes and extracted from the VDiagnosis, InpatientDiagnosis, PatientTransferDiagnosis, and SpecialtyTransferDiagnosis tables in inpatient and outpatient domains within CDW. Asthma was defined by ICD-10 codes J45* and J46*; COPD by J41*, J42*, J43*, and J44*; and pneumonia by J09.X1, J10*-J18*, A01.03, A02.22, A37.01, A37.11, A37.81, A37.91, A54.84, B01.2, B05.2, B06.81, B77.81, J85.1, and J22*.

Combat Veteran Status

Combat status for veterans in our cohort was extracted from several domains and data sources in CDW, including the SPatientDisability, HealthFactor, Visit, Purple Heart, and RxOutPat tables from the Patient, HF, Outpat, and RxOutpat tables, respectively, as well as the United States Veterans Eligibility Trends and Statistics (US VETS) data source. Combat status records were then linked to service and deployment records in the PeriodOfService and SPatientDisability tables to identify the associated combat era and geographic region of deployment.

Albuterol Fills

Albuterol fills were extracted from CDW's outpatient pharmacy domain in the RxOutpatFill table using the date and time that albuterol medications were filled (ReleaseDateTime) and the quantity dispensed (QtyNumeric). We used inhaler equivalents to standardize rescue inhaler doses between inhalers and nebulizers. The inhaler equivalents of nebulizers were calculated by dividing the quantity of vials in each nebulizer prescription by 100. For example, a prescription for a nebulizer with a quantity of 120 vials was converted to an albuterol inhaler equivalent of 1.2. Prescriptions for inhalers had an albuterol inhaler equivalent that was the quantity of inhalers dispensed for that prescription.

Prednisone Fills

Prednisone fills were extracted from the RxOutpatFill table in the pharmacy domain. The ReleaseDateTime was used to identify prednisone fills per patient day during the study period.

Emergency Department (ED) Visits

ED visits were obtained from the Emergency Department Integration Software (EDIS) and Fee_Based domains in CDW, the Integrated Veteran Care (IVC) Consolidated Data Sets (CDS), and the Program Integrity Tool (PIT). Data from the US Centers for Medicare & Medicaid Services (CMS) were used to capture CMS ED visits by matching scrambled Social Security numbers from CMS data to those in CDW. The VA, Fee, and CMS ED visit datasets were combined into a single table (N=230,006) with one row per ED visit that contained the date of visit, diagnosis indicators for COPD, asthma, and

pneumonia, and the presence or absence of an associated hospitalization, defined as a hospitalization within 1 calendar day of an ED visit. ED visits that occurred within 1 calendar day of a preceding ED visit were dropped.

Hospitalizations

VA hospitalizations were obtained from the Inpatient domain in CDW, while non-VA (community care) hospitalizations were obtained from the Fee_Based domain, IVC-CDS, and PIT data sources. CMS data were also used to identify hospitalizations in the Medicare Provider Analysis and Review (MedPAR) dataset. Community care hospitalizations found in CDS, PIT, and CDW-Fee schemas were combined with the VA-based and CMS hospitalizations (N=210,263). The hospitalization table contained dates of hospital admission and discharge, diagnosis indicators for COPD, asthma, and pneumonia, and hospital length of stay. Unique hospitalizations occurring within 1 calendar day were merged, with subsequent hospitalizations grouped under the initial admission.

Inhaler-Related Greenhouse Gas Emissions Estimates

Inhaler-related emissions were estimated using previously reported values for the budesonide–formoterol metered-dose inhaler (38.2 kg of carbon dioxide equivalent [CO_2e] per inhaler) and the fluticasone–salmeterol dry-powder inhaler (0.898 kg of CO_2e per inhaler).[2] Inhaler-related emissions of other combination controller inhalers included in the study (41.4 kg of CO_2e per inhaler) were calculated using a weighted estimate of the non-budesonide–formoterol and non-fluticasone–salmeterol inhalers filled during the study period.[2,3] To estimate annual inhaler-related emissions, the mean emissions per inhaler were multiplied by the number of yearly prescription fills for each category of inhaler using the total study cohort (N=347,486).

© 2025 Rabin AS et al. *JAMA Intern Med*.

eTable 1. Matched Observational Cohort Study Design

Protocol Element	Description	Implementation
Eligibility criteria	Who will be included in this study?	Patients meeting these criteria:<br>1. Dispensed a budesonide–formoterol MDI controller inhaler in the 180 days prior to the national formulary change (180 days leading up to and including 6/30/2021)<br>2. Not dispensed fluticasone–salmeterol DPI in the 180 days prior to the national formulary change (180 days leading up to and including 6/30/2021)<br>3. Dispensed either a fluticasone–salmeterol DPI inhaler or a non-fluticasone-salmeterol DPI in the 180 days following the formulary change (180 days including and following 7/1/2021)
Treatment strategies	Which precise treatment strategies or interventions will eligible individuals receive?	1. Transition to fluticasone–salmeterol DPI: dispensed a fluticasone–salmeterol DPI in the 180 days following formulary change (180 days including and following 7/1/2021)<br>2. Continuation of non-fluticasone–salmeterol DPI: dispensed a non-fluticasone–salmeterol DPI the 180 days following the national formulary change (180 days including and following 7/1/2021) AND not dispensed fluticasone-salmeterol DPI
Treatment assignment	How will eligible individuals be assigned to the treatment strategies?	1. All patients dispensed a fluticasone–salmeterol dry-powder inhaler in the 180 days following the national formulary change are assigned to the switched group<br>2. Patients dispensed a non-fluticasone–salmeterol dry-powder inhaler AND NOT dispensed fluticasone–salmeterol dry-powder inhaler during the 180 days following the formulary transition are assigned to the not-switched group
Outcomes	What outcomes will be measured during follow-up?	The following outcomes are measured at 90 and 180 days from the enrollment date.<br>1. Mortality<br>2. Albuterol fills (count)<br>3. Prednisone fills (count)<br>4. ED visits (4a. all-cause, 4b. respiratory, 4c. pneumonia)<br>5. Hospitalizations (5a. all-cause, 5b. respiratory, 5c. pneumonia)
Enrollment date	When are patients enrolled?	1. Switched group: First date for fluticasone–salmeterol DPI fill including and following July 1, 2021<br>2. Not-switched group: First date for non-fluticasone–salmeterol DPI fill including and following July 1, 2021
Causal estimand	Which causal estimand will be estimated with the observational data?	Intention-to-treat (inhaler transitions after enrollment are not factored into the analysis)
Start and end of follow-up	When does follow-up start, and when does it end?	Follow-up starts on the day of enrollment (day 1) and ends at day 180
Statistical analysis	Which statistical analyses will be used to estimate the causal estimand?	We used a two-step procedure to reduce confounding:<br>1. Weighting with CEM weights to balance budesonide-formoterol MDI and fluticasone–salmeterol DPI groups on each of the following variables measured at enrollment: age, sex, asthma diagnosis, COPD diagnosis, hospitalization in prior year (Y/N), ED visit in prior year (Y/N), number of albuterol fills in prior year (0-1, 2, 3+), prednisone in prior year (Y/N), smoking status (current, prior, never), and geographic region at time of enrollment.<br>2. Regression adjustment to provide secondary control of confounding and precise estimates of effect. We used multivariable logistic regression to calculate adjusted odds ratios (and margins) for binary outcomes and Poisson regression to calculate adjusted risk ratios for continuous outcomes.<br>Statistical code is available at GitHub (https://github.com/CCMRPulmCritCare/VA_SWITCH)
Prespecified sensitivity analyses		No, because the self-controlled case series is the primary analysis, and the matched observational cohort study is the secondary analysis

Abbreviations: CEM, coarsened exact matching; COPD, chronic obstructive pulmonary disease; DPI, dry-powder inhaler; ED, emergency department; MDI, metered-dose inhaler.

© 2025 Rabin AS et al. *JAMA Intern Med.*

eTable 2. Total Exposure Time and Outcomes During Exposure Periods[a]

	Budesonide–Formoterol Metered-Dose Inhaler	Fluticasone–Salmeterol Dry-Powder Inhaler
Total exposure in person-years	485,695	174,814
Total albuterol fills	2,982,408	1,029,221
Total prednisone fills	210,051	77,821
Emergency department visits	261,853	115,921
Hospitalizations	171,635	89,149

[a] Total exposure time presented using the 33% grace period approach, as presented in the self-controlled case series analysis.

© 2025 Rabin AS et al. *JAMA Intern Med*.

eTable 3. Sensitivity Analysis of Fluticasone–Salmeterol Dry-Powder Inhaler vs Budesonide–Formoterol Metered-Dose Inhaler on Clinical Outcomes by Approach to Operationalizing Inhaler Exposure Periods[a]

A. Albuterol Fills

Adherence	Exclusion Criteria	No. of Patients	No. (%) of Patients with Albuterol Fills	Incidence Rate Ratio (95% CI)[b]	P Value
Strict	none	260,268	230,492 (88.6)	0.90 (0.90, 0.90)	<0.001
Strict	>3 month gap	14,646	13,463 (91.9)	0.96 (0.94, 0.97)	<0.001
Plus 33%	none	259,602	231,403 (89.1)	0.90 (0.90, 0.91)	<0.001
Plus 33%	>3 month gap	42,497	39,131 (92.1)	0.96 (0.95, 0.97)	<0.001
Plus 100%	none	258,522	232,104 (89.8)	0.93 (0.92, 0.93)	<0.001
Plus 100%	>3 month gap	84,837	78,150 (92.1)	0.96 (0.96, 0.97)	<0.001

B. Prednisone Courses

Adherence	Exclusion Criteria	No. of Patients	No. (%) of Patients with Prednisone Fills	Incidence Rate Ratio (95% CI)[b]	P Value
Strict	none	260,268	95,215 (36.6)	1.01 (1.00, 1.02)	0.135
Strict	>3 month gap	14,646	5,149 (35.2)	1.04 (0.99, 1.10)	0.134
Plus 33%	none	259,602	94,926 (36.6)	1.02 (1.01, 1.03)	0.002
Plus 33%	>3 month gap	42,497	15,138 (35.6)	1.03 (1.00, 1.07)	0.040
Plus 100%	none	258,522	94,395 (36.5)	1.03 (1.02, 1.05)	<0.001
Plus 100%	>3 month gap	84,837	30,797 (36.3)	1.04 (1.01, 1.06)	0.002

C. All-Cause ED Visit

Adherence	Exclusion Criteria	No. of Patients	No. (%) of Patients with All-Cause ED Visit	Incidence Rate Ratio (95% CI)[b]	P Value
Strict	none	260,268	157,876 (60.7)	1.05 (1.04, 1.06)	<0.001
Strict	>3 month gap	14,646	8,351 (57.0)	1.12 (1.06, 1.18)	<0.001
Plus 33%	none	259,602	157,442 (60.6)	1.05 (1.04, 1.06)	<0.001
Plus 33%	>3 month gap	42,497	24,718 (58.2)	1.10 (1.06, 1.13)	<0.001
Plus 100%	none	258,522	156,780 (60.6)	1.05 (1.04, 1.07)	<0.001
Plus 100%	>3 month gap	84,837	50,312 (59.3)	1.07 (1.05, 1.09)	<0.001

D. Respiratory-Related ED Visit

Adherence	Exclusion Criteria	No. of Patients	No. (%) of Patients with Respiratory ED Visit	Incidence Rate Ratio (95% CI)[b]	P Value
Strict	none	260,268	43,241 (16.6)	1.05 (1.02, 1.08)	0.001
Strict	>3 month gap	14,646	2,513 (17.2)	1.15 (1.01, 1.31)	0.030
Plus 33%	none	259,602	43,100 (16.6)	1.06 (1.03, 1.09)	<0.001
Plus 33%	>3 month gap	42,497	7,702 (18.1)	1.18 (1.09, 1.27)	<0.001
Plus 100%	none	258,522	42,864 (16.6)	1.06 (1.03, 1.09)	<0.001
Plus 100%	>3 month gap	84,837	15,330 (18.1)	1.14 (1.08, 1.21)	<0.001

E. Pneumonia-Related ED Visit

Adherence	Exclusion Criteria	No. of Patients	No. (%) of Patients with Pneumonia ED Visit	Incidence Rate Ratio (95% CI)[b]	P Value
Strict	none	260,268	15,205 (5.8)	1.24 (1.17, 1.31)	<0.001
Strict	>3 month gap	14,646	818 (5.6)	1.00 (0.76, 1.32)	0.978
Plus 33%	none	259,602	15,139 (5.8)	1.25 (1.18, 1.32)	<0.001
Plus 33%	>3 month gap	42,497	2,665 (6.3)	1.28 (1.09, 1.50)	0.003
Plus 100%	none	258,522	15,041 (5.8)	1.25 (1.17, 1.33)	<0.001
Plus 100%	>3 month gap	84,837	5,399 (6.4)	1.28 (1.14, 1.43)	<0.001

© 2025 Rabin AS et al. *JAMA Intern Med.*

F. All-Cause Hospitalization

Adherence	Exclusion Criteria	No. of Patients	No. (%) of Patients with All-Cause Hospitalization	Incidence Rate Ratio (95% CI)[b]	P Value
Strict	none	260,268	139,946 (53.8)	1.08 (1.07, 1.09)	<0.001
Strict	>3 month gap	14,646	7,500 (51.2)	1.10 (1.03, 1.17)	0.003
Plus 33%	none	259,602	139,590 (53.8)	1.08 (1.06, 1.09)	<0.001
Plus 33%	>3 month gap	42,497	22,555 (53.1)	1.13 (1.09, 1.17)	<0.001
Plus 100%	none	258,522	138,987 (53.8)	1.08 (1.06, 1.09)	<0.001
Plus 100%	>3 month gap	84,837	46,140 (54.4)	1.12 (1.09, 1.15)	<0.001

G. Respiratory-Related Hospitalization

Adherence	Exclusion Criteria	No. of Patients	No. (%) of Patients with Respiratory Hospitalization	Incidence Rate Ratio (95% CI)[b]	P Value
Strict	none	260,268	37,445 (14.4)	1.10 (1.07, 1.13)	<0.001
Strict	>3 month gap	14,646	2,259 (15.4)	1.18 (1.02, 1.36)	0.025
Plus 33%	none	259,602	37,331 (14.4)	1.10 (1.07, 1.14)	<0.001
Plus 33%	>3 month gap	42,497	6,956 (16.4)	1.22 (1.13, 1.33)	<0.001
Plus 100%	none	258,522	37,115 (14.4)	1.11 (1.07, 1.15)	<0.001
Plus 100%	>3 month gap	84,837	13,988 (16.5)	1.19 (1.12, 1.26)	<0.001

H. Pneumonia-Related Hospitalization

Adherence	Exclusion Criteria	No. of Patients	No. (%) of Patients with Pneumonia Hospitalization	Incidence Rate Ratio (95% CI)[b]	P Value
Strict	none	260,268	16,904 (6.5)	1.25 (1.18, 1.32)	<0.001
Strict	>3 month gap	14,646	1,029 (7.0)	1.26 (0.97, 1.62)	0.079
Plus 33%	none	259,602	16,855 (6.5)	1.24 (1.17, 1.31)	<0.001
Plus 33%	>3 month gap	42,497	3,146 (7.4)	1.32 (1.14, 1.53)	<0.001
Plus 100%	none	258,522	16,747 (6.5)	1.24 (1.17, 1.32)	<0.001
Plus 100%	>3 month gap	84,837	6,332 (7.5)	1.32 (1.19, 1.46)	<0.001

Abbreviation: ED, emergency department.

[a] Shown are sensitivity analyses for variable exposure models of medication adherence, termed strict, plus 33%, or plus 100%. Strict adherence was defined as receipt of the controller inhaler only for the duration prescribed (eg, a 30-day fill would equate to 30 days of treatment). Plus 33% adherence was defined as receipt of the controller inhaler for 133% of the duration prescribed (eg, a 30-day fill would equate to 40 days of treatment). Plus 100% adherence was defined as receipt of the controller inhaler for 200% of the duration prescribed (eg, a 30-day fill would equate to 60 days of treatment). For each exposure model, additional sensitivity analyses were performed to exclude patients with a greater than 3-month gap in controller inhaler treatment. Only patients experiencing the specified outcome of interest (eg, patients dispensed an albuterol inhaler) were included in each model.

[b] Value greater than 1 favors budesonide–formoterol metered-dose inhaler, and a value less than 1 favors fluticasone–salmeterol dry-powder inhaler.

© 2025 Rabin AS et al. *JAMA Intern Med*.

eTable 4. Sensitivity Analysis Excluding Emergency Department (ED) Visits Occurring Near to Hospitalization for Pneumonia[a]

**Outcome**	**No. of Patients**	**Incidence Rate Ratio (95% CI)**	**P Value**
Pneumonia ED visit			
Primary Analysis	15,139	1.25 (1.18, 1.32)	<0.001
Excluding pneumonia ED visit + pneumonia hospitalization	9,287	1.21 (1.12, 1.31)	<0.001

[a] Sensitivity analysis showing the exclusion of patients who had an ED visit for pneumonia on the same day or the day prior to a hospitalization.

© 2025 Rabin AS et al. *JAMA Intern Med*.

eTable 5. Sensitivity Analysis Examining Patients With 1 vs 2+ Events During the Study Period

	**All Patients (Primary Analysis)**		**1 event (Sensitivity Analysis)**		**2+ events (Sensitivity Analysis)**	
	No.	IRR	No.	IRR	No.	IRR
Albuterol fills	231,403	0.90	--	--	231,403	0.90
Prednisone courses	94,926	1.02	38,778	0.95	56,148	1.03
All-cause ED visit	157,442	1.05	50,549	1.03	106,893	1.05
Respiratory ED visit	43,100	1.06	27,365	1.02	15,735	1.07
Pneumonia ED visit	15,139	1.25	12,695	1.22	2,444	1.32
All-cause hospitalization	139,590	1.08	55,596	1.09	83,994	1.08
Respiratory hospitalization	259,602	1.10	24,741	1.09	12,590	1.11
Pneumonia hospitalization	259,602	1.24	5,562	1.24	1,702	1.23

Abbreviations: ED, emergency department; IRR, incidence rate ratio.

© 2025 Rabin AS et al. *JAMA Intern Med*.

eTable 6. Ratio of Forced Expiratory Volume in 1 Second (FEV_1) to Forced Vital Capacity (FVC) and FEV_1 Percent Predicted in the Study Cohort[a]

Variable	Value
FEV_1:FVC ratio — no. (%)	
Ratio <70% of predicted	7,315 (72.6)
Ratio ≥70% of predicted	2,747 (27.3)
FEV_1 — no. (% of the predicted value) — no. (%)	
Normal (≥80% of predicted)	1,823 (21.2)
Mildly reduced (70-79% of predicted)	1,353 (15.7)
Moderately reduced (60-69% of predicted)	1,528 (17.8)
Moderately severely reduced (50-59% of predicted)	1,026 (11.9)
Severely reduced (35%-49% of predicted)	1,958 (22.8)
Very severely reduced (<35% of predicted)	902 (10.5)

[a] Values of FEV_1:FVC and FEV_1 results were available for 10,062 and 8,590 patients, respectively. Data were extracted from the electronic health record, as previously reported.[4]

© 2025 Rabin AS et al. *JAMA Intern Med.*

eTable 7. Impact of Fluticasone–Salmeterol Dry-Powder Inhaler vs Budesonide–Formoterol Metered-Dose Inhaler on Clinical Outcomes by Forced Expiratory Volume in 1 Second (FEV_1) Percent Predicted[a]

Outcome	No. of Patients	Incidence Rate Ratio (95% CI)[b]	P Value
Albuterol fills			
FEV_1 ≥50% of predicted	5,329	0.88 (0.86, 0.90)	<0.001
FEV_1 <50% of predicted	2,793	0.92 (0.89, 0.94)	<0.001
Prednisone fills			
FEV_1 ≥50% of predicted	2,968	0.98 (0.91, 1.05)	0.548
FEV_1 <50% of predicted	1,855	0.92 (0.86, 0.99)	0.037
All-cause ED visit			
FEV_1 ≥50% of predicted	3,602	0.98 (0.92, 1.05)	0.604
FEV_1 <50% of predicted	1,967	1.12 (1.03, 1.22)	0.011
Respiratory ED visit			
FEV_1 ≥50% of predicted	987	1.13 (0.93, 1.37)	0.212
FEV_1 <50% of predicted	946	1.15 (0.96, 1.38)	0.134
Pneumonia ED visit			
FEV_1 ≥50% of predicted	328	1.47 (0.95, 2.27)	0.086
FEV_1 <50% of predicted	295	1.65 (1.06, 2.56)	0.027
All-cause hospitalization			
FEV_1 ≥50% of predicted	3,412	1.00 (0.93, 1.08)	0.900
FEV_1 <50% of predicted	2,086	1.06 (0.97, 1.16)	0.192
Respiratory hospitalization			
FEV_1 ≥50% of predicted	823	1.10 (0.89, 1.35)	0.380
FEV_1 <50% of predicted	1,009	1.05 (0.88, 1.25)	0.590
Pneumonia hospitalization			
FEV_1 ≥50% of predicted	-	Did not converge	-
FEV_1 <50% of predicted	-	Did not converge	-

Abbreviation: ED, emergency department.

[a] When more than one value of FEV_1 was available, the first recorded value during the study period was used.

[b] Value greater than 1 favors budesonide–formoterol metered-dose inhaler and a value less than 1 favors fluticasone–salmeterol dry-powder inhaler.

© 2025 Rabin AS et al. *JAMA Intern Med.*

eTable 8. Estimated Inhaler-Related Greenhouse Gas (GHG) Emissions in the Study Cohort[a]

Year	No. of Budesonide–Formoterol Metered-Dose Inhalers Filled	No. of Fluticasone–Salmeterol Dry-Powder Inhalers Filled	No. of Other Controller Inhalers Filled	Estimated Controller Inhaler-Related Emissions in MMT of CO_2e	Estimated Controller Inhaler-Related Emissions per Veteran in kg of CO_2e[b]
2018	1,362,791	0	173,705	0.059	279.78
2019	1,564,172	14,310	241,146	0.070	280.46
2020	1,825,628	36,766	316,958	0.083	293.52
2021[c]	1,413,212	704,281	435,952	0.073	221.01
2022	194,081	1,560,725	573,363	0.033	101.04

Abbreviations: CO_2e, carbon dioxide equivalent; MMT, million metric tons.

[a] The total estimated reduction in controller inhaler-related GHG emissions from 2018 to 2022 of 0.027 MMT of CO_2e was equivalent to the emissions of approximately 6,229 gasoline-powered passenger vehicles driven for 1 year.[5]

[b] 10 kg of CO_2e equals approximately 41 km driven in an average gasoline-powered passenger vehicle.[5]

[c] The formulary change from the budesonide–formoterol metered-dose inhaler to the fluticasone–salmeterol dry-powder inhaler occurred in July 2021.

eTable 9. Covariate Balance of Switched vs Not-Switched Populations in the Matched Observational Cohort Study[a]

	Switched Patients Included in Analysis (N=167,331)	Not-Switched Patients Included in Analysis (N=91,226)	Standardized Mean Difference of Total Unweighted Switched and Not-Switched Populations (N=263,079)	Standardized Mean Difference of Weighted Populations Included in Analysis (N=258,557)
Age in years, mean	68.9	68.9	0.121	0.001
Male sex, %	93.7	93.7	0.045	<0.001
Asthma diagnosis, %	29.8	29.8	0.114	<0.001
COPD diagnosis, %	73.1	73.1	0.109	<0.001
Hospitalization in prior year, %	22.3	22.3	0.029	<0.001
Emergency department visit in prior year, %	27.7	27.7	0.011	<0.001
Albuterol fills in prior year, %			0.112	<0.001
0-1	24.3	24.1		
2	8.2	8.4		
3+	67.4	67.4		
Prednisone course in prior year, %	14.7	14.7	0.018	<0.001
Smoking status, %			0.072	<0.001
Current	28.3	27.3		
Former	54.1	55.1		
Never	17.6	17.6		
Geographic region, %			0.255	<0.001
Northwest	12.7	10.8		
Midwest	23.9	25.9		
South	45.7	45.7		
West	17.6	17.6		

Abbreviation: COPD, chronic obstructive pulmonary disease.

[a] Patients who switched were transitioned from a budesonide–formoterol metered-dose inhaler to a fluticasone–salmeterol dry-powder inhaler. Patients who were not switched continued on a non-fluticasone–salmeterol dry-powder inhaler. Additional details provided in **eTable 1**.

eTable 10. Estimated 90-Day Health Outcomes (Adjusted) of Switched vs Not-Switched Patients in a Matched Observational Cohort Study

**Outcome**[a]	**Adjusted Count of Switched (N=164,235)**	**Adjusted Count of Not-Switched (N=102,093)**	**Incident Rate Ratio (95% CI)**	**Adjusted Absolute Difference**
Albuterol fills[b]	1.90	1.92	0.99 (0.97, 1.02)	-0.02 (-0.06, 0.03)
Prednisone fills[c]	0.11	0.11	1.00 (0.97, 1.03)	0.0001 (-0.003, 0.004)
	**Adjusted Proportion of Switched (N=164,235)**	**Adjusted Proportion of Not-Switched (N=102,093)**	**Adjusted Odds Ratio (95% CI)**	**Adjusted Absolute Difference**
All-cause ED visit	11.13%	10.31%	1.10 (1.07, 1.14)	0.82% (0.57%, 1.06%)
Respiratory ED visit	1.63%	1.48%	1.11 (1.04, 1.19)	0.16% (0.05%, 0.26%)
Pneumonia ED visit	0.44%	0.37%	1.17 (1.03, 1.34)	0.06% (0.01%, 0.11%)
All-cause hospitalization	8.87%	8.54%	1.05 (1.02, 1.08)	0.34% (0.11%, 0.56%)
Respiratory hospitalization	1.56%	1.34%	1.17 (1.09, 1.26)	0.22% (0.12%, 0.32%)
Pneumonia hospitalization	0.52%	0.45%	1.18 (1.04, 1.33)	0.08% (0.02%, 0.13%)
Mortality	0.46%	0.52%	0.89 (0.80, 1.01)	-0.05% (-0.11%, 0.004%)

Abbreviation: ED, emergency department.

[a] Outcomes were estimated using logistic or Poisson regression with weights generated from coarsened exact matching to balance the following variables between switched and not switched veterans: age, sex, respiratory diagnosis, health care utilization, and medication fills in the year prior to inhaler switch, smoking status, and geographic region. Additionally, these variables were included as covariates in the regression models. Predictive margins are reported for each treatment group.

[b] Albuterol represented as inhaler-equivalent medication fills.

[c] Prednisone represented as discrete courses of the medication filled.

© 2025 Rabin AS et al. *JAMA Intern Med.*

eTable 11. Estimated 180-Day Health Outcomes (Unadjusted) of Switched vs Not-Switched Patients in the Matched Observational Cohort Study

**Outcome**[a]	**Count of Switched (N=162,928)**	**Count of Not-Switched (N=89,643)**	**Odds Ratio (95% CI)**	**Absolute Difference (95% CI)**
Albuterol fills[b]	3.47	3.42	1.01 (0.99, 1.04)	0.05 (-0.03, 0.13)
Prednisone fills[c]	0.21	0.21	1.01 (0.98, 1.04)	0.001 (-0.005, 0.008)
	**Proportion of Switched (N=162,928)**	**Proportion of Not-Switched (N=89,643)**	**Odds Ratio (95% CI)**	**Absolute Difference (95% CI)**
All-cause ED visit	19.46%	18.30%	1.08 (1.05, 1.10)	1.15% (0.82%, 1.49%)
Respiratory ED visit	3.29%	2.97%	1.11 (1.06, 1.17)	0.32% (0.17%, 0.47%)
Pneumonia ED visit	0.95%	0.84%	1.14 (1.04, 1.25)	0.12% (0.04%, 0.19%)
All-cause hospitalization	16.15%	15.63%	1.04 (1.02, 1.06)	0.52% (0.21%, 0.83%)
Respiratory hospitalization	3.16%	2.74%	1.16 (1.10, 1.22)	0.42% (0.28%, 0.56%)
Pneumonia hospitalization	1.15%	1.03%	1.12 (1.03, 1.21)	0.12% (0.04%, 0.21%)
Mortality	1.89%	1.90%	0.99 (0.93, 1.06)	-0.01% (-0.12%, 0.10%)

Abbreviation: ED, emergency department.

[a] Outcomes were estimated using logistic or Poisson regression with weights generated from coarsened exact matching. Model weighting was performed using variables for age, sex, respiratory diagnosis, health care utilization, and medication fills in the year prior to inhaler switch, smoking status, and geographic region. Predictive margins are reported for each treatment group.

[b] Albuterol represented as inhaler-equivalent medication fills.

[c] Prednisone represented as discrete courses of the medication filled.

© 2025 Rabin AS et al. *JAMA Intern Med.*

eTable 12. Estimated 90-Day Health Outcomes (Unadjusted) of Switched vs Not-Switched Patients in the Matched Observational Cohort Study

Outcome[a]	Count of Switched (N=162,928)	Count of Not-Switched (N=89,643)	Odds Ratio (95% CI)	Absolute Difference (95% CI)
Albuterol fills[b]	1.90	1.92	0.99 (0.97, 1.02)	-0.02 (-0.06, 0.03)
Prednisone fills[c]	0.11	0.11	1.00 (0.96, 1.04)	-0.0001 (-0.004, 0.004)
	**Proportion of Switched (N=162,928)**	**Proportion of Not-Switched (N=89,643)**	**Odds Ratio (95% CI)**	**Absolute Difference (95% CI)**
All-cause ED visit	11.13%	10.30%	1.09 (1.06, 1.12)	0.83% (0.57%, 1.09%)
Respiratory ED visit	1.63%	1.47%	1.11 (1.04, 1.19)	0.16% (0.05%, 0.26%)
Pneumonia ED visit	0.43%	0.38%	1.16 (1.02, 1.33)	0.06% (0.01%, 0.11%)
All-cause hospitalization	8.89%	8.53%	1.05 (1.01, 1.08)	0.35% (0.12%, 0.59%)
Respiratory hospitalization	1.56%	1.34%	1.17 (1.09, 1.26)	0.22% (0.12%, 0.32%)
Pneumonia hospitalization	0.52%	0.45%	1.17 (1.04, 1.32)	0.08% (0.02%, 0.13%)
Mortality	0.46%	0.52%	0.89 (0.80, 1.01)	-0.05% (-0.11%, 0.005%)

Abbreviation: ED, emergency department.

[a] Outcomes were estimated using logistic or Poisson regression with weights generated from coarsened exact matching. Model weighting was performed using variables for age, sex, respiratory diagnosis, health care utilization, and medication fills in the year prior to inhaler switch, smoking status, and geographic region. Predictive margins are reported for each treatment group.

[b] Albuterol represented as inhaler-equivalent medication fills.

[c] Prednisone represented as discrete courses of the medication filled.

© 2025 Rabin AS et al. *JAMA Intern Med.*

eFigure 1. Three Approaches to Operationalizing Inhaler Exposure Periods: No Grace Period, 33% Grace Period, and 100% Grace Period

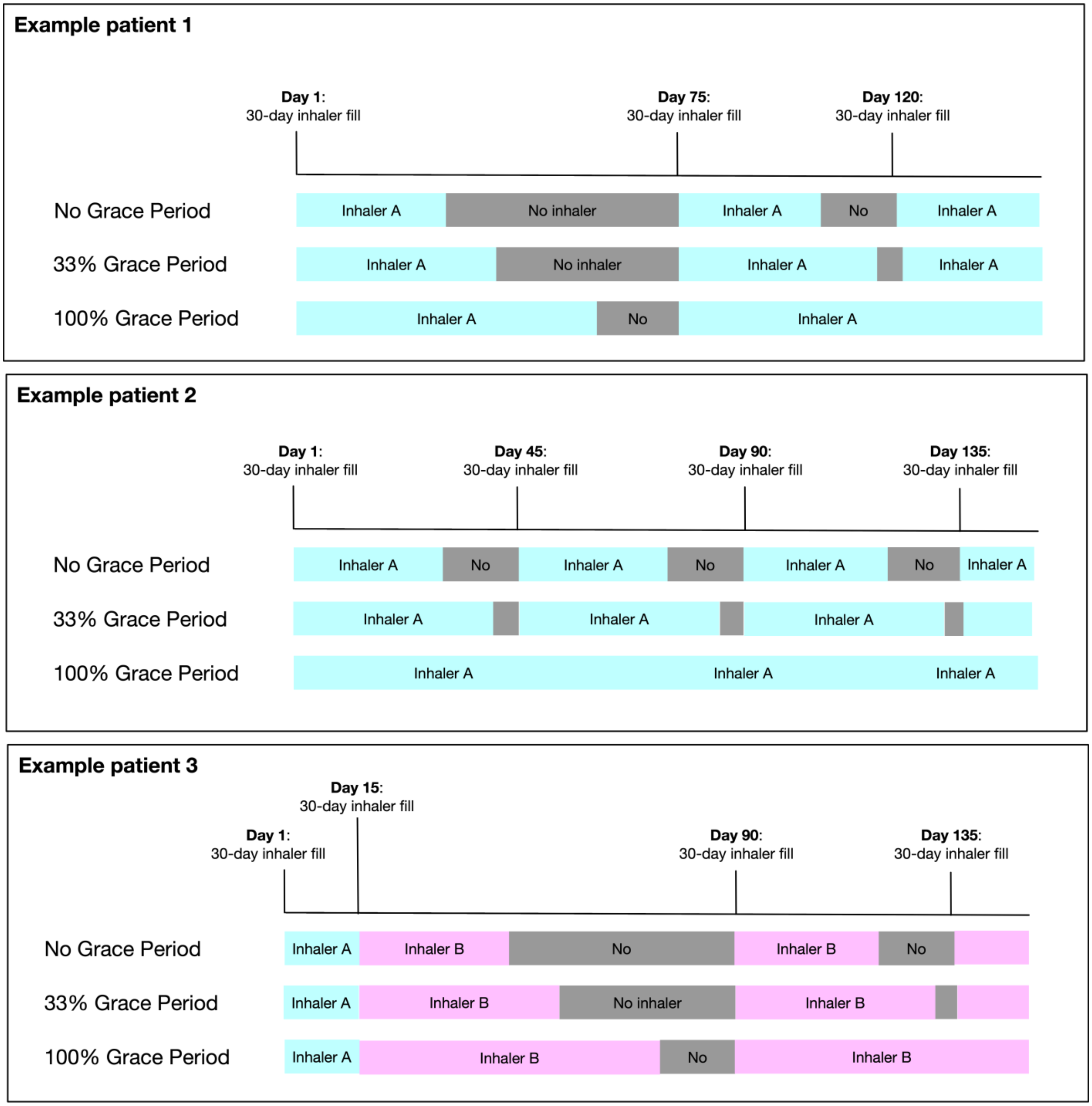

Shown are inhaler refill timelines for three hypothetical patients. We considered three approaches: no grace period, a 33% grace period (primary analysis), and a 100% grace period. In the no grace period model, patients were considered on controller inhalers only for the number of days dispensed (ie, patients were considered to be taking 100% of their prescribed doses). In the 33% grace period models, patients were considered to be on inhalers for an additional 33% of days (ie, 40 days for a 30-day fill; the model therefore assumes a patient is taking 75% of their prescribed doses). In the 100% grace period model, patients were considered to be on an inhaler for an additional 100% of days (ie, 60 days for a 30-day fill; the model therefore assumes a patient is taking 50% of their prescribed doses). In all models, subsequent prescriptions take precedence over the prior prescription.

© 2025 Rabin AS et al. *JAMA Intern Med.*

eFigure 2. Patients Included in the Study Cohort

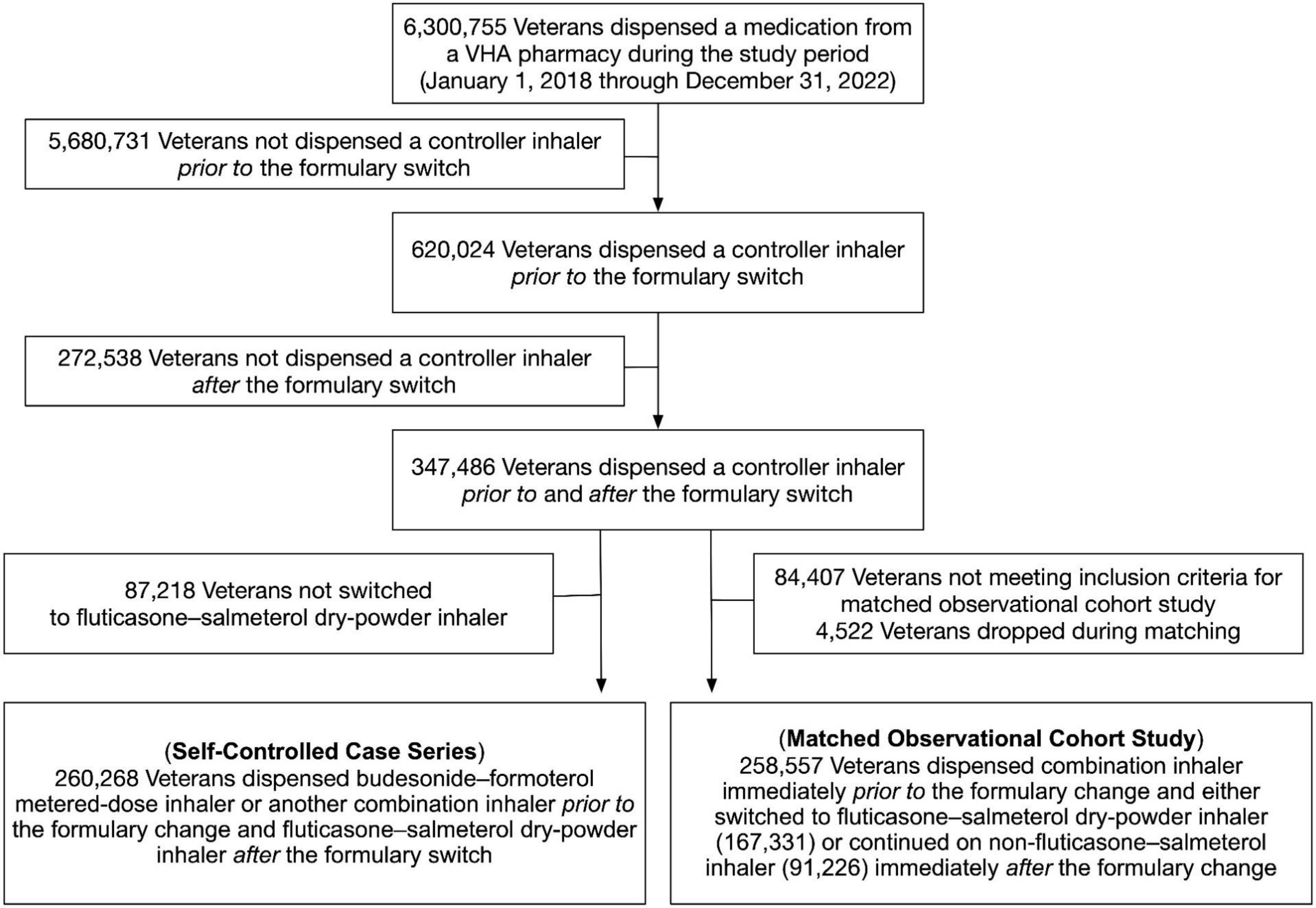

VHA indicates Veterans Health Administration. Shown is the selection of patients for the self-controlled case series and the matched observational cohort study.

© 2025 Rabin AS et al. *JAMA Intern Med*.

eFigure 3. Clinical Outcomes Stratified by Subgroups

Panel A

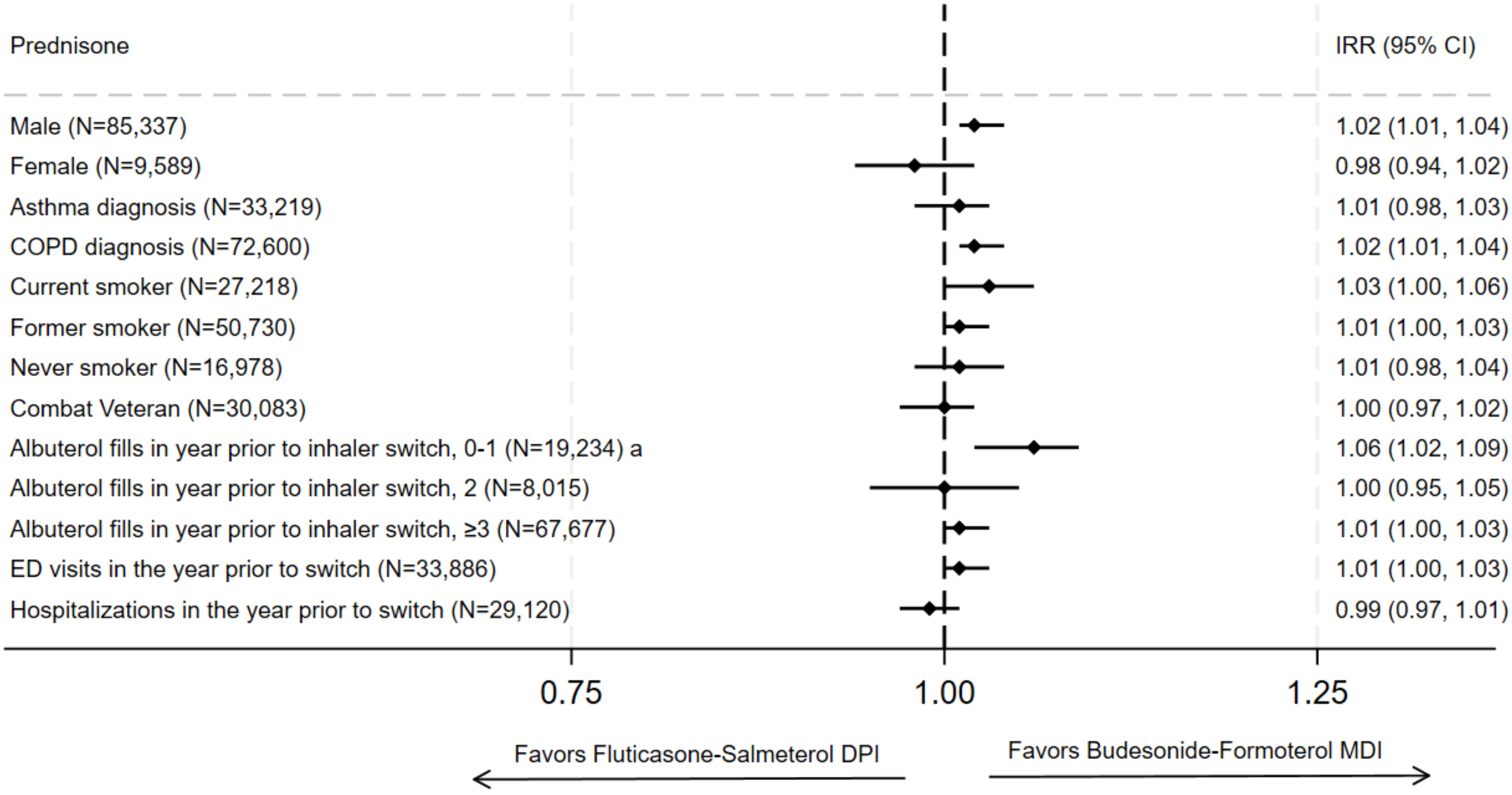

© 2025 Rabin AS et al. *JAMA Intern Med.*

Panel B

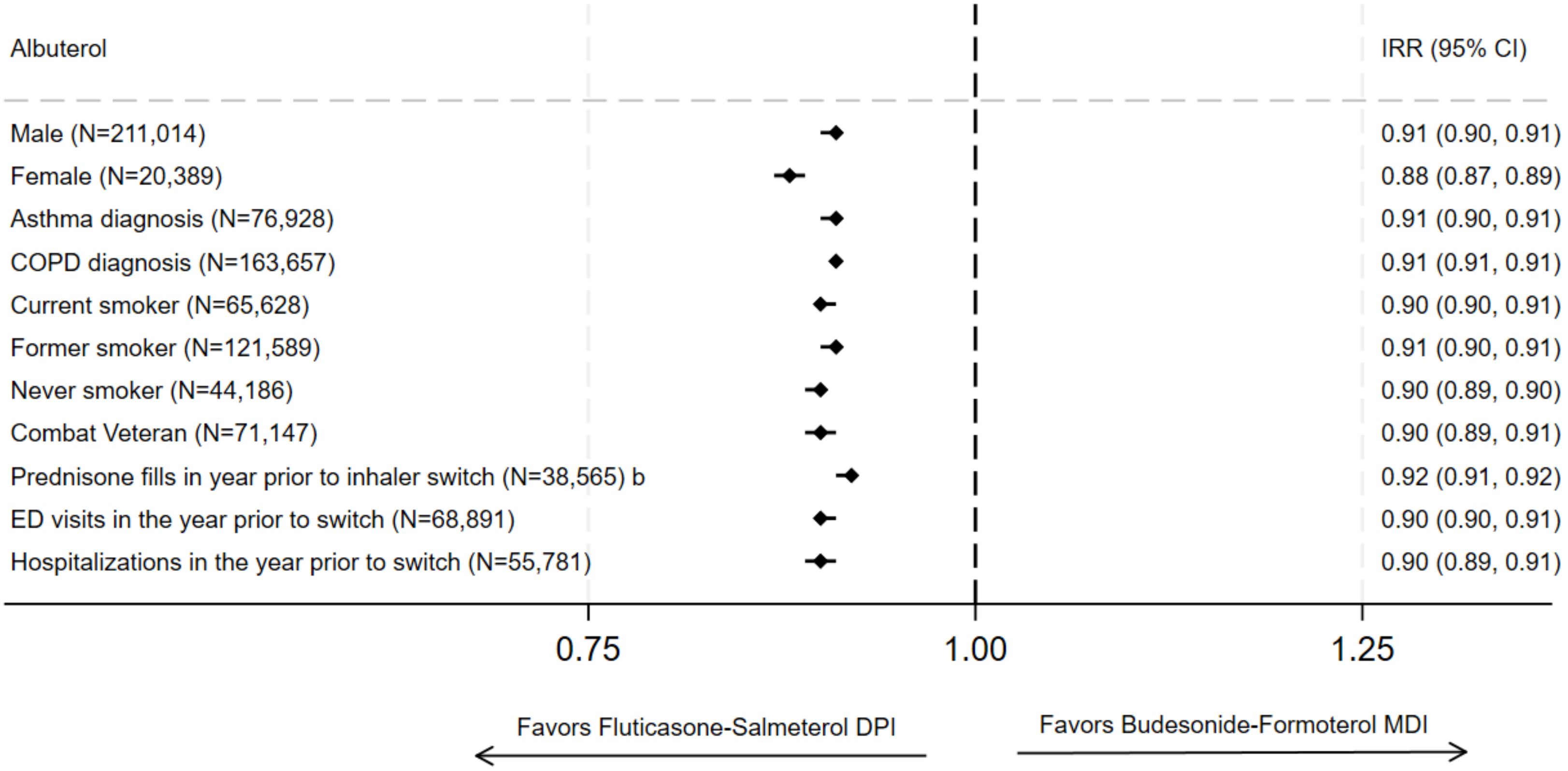

© 2025 Rabin AS et al. *JAMA Intern Med*.

Panel C

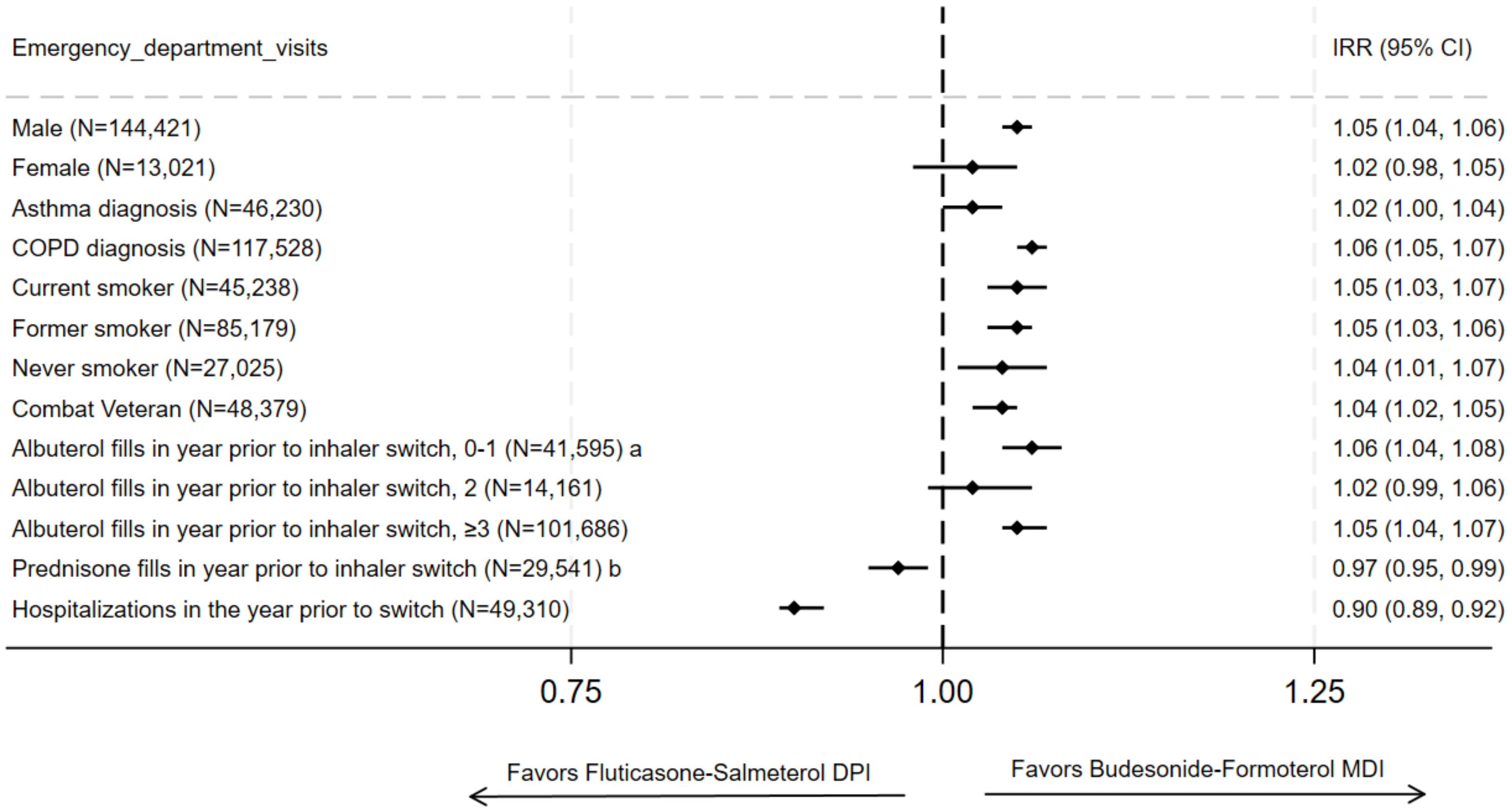

© 2025 Rabin AS et al. *JAMA Intern Med*.

Panel D

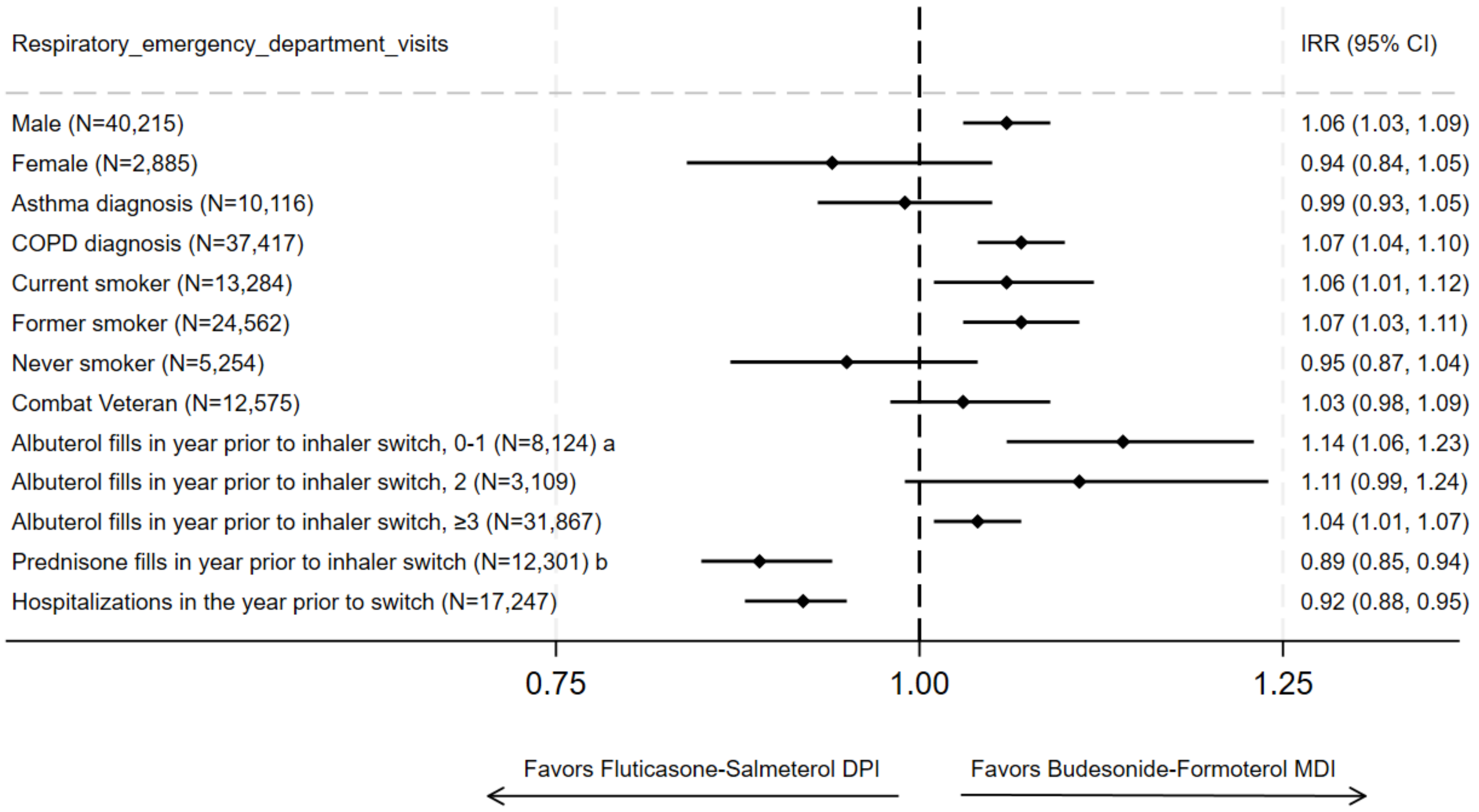

© 2025 Rabin AS et al. *JAMA Intern Med*.

Panel E

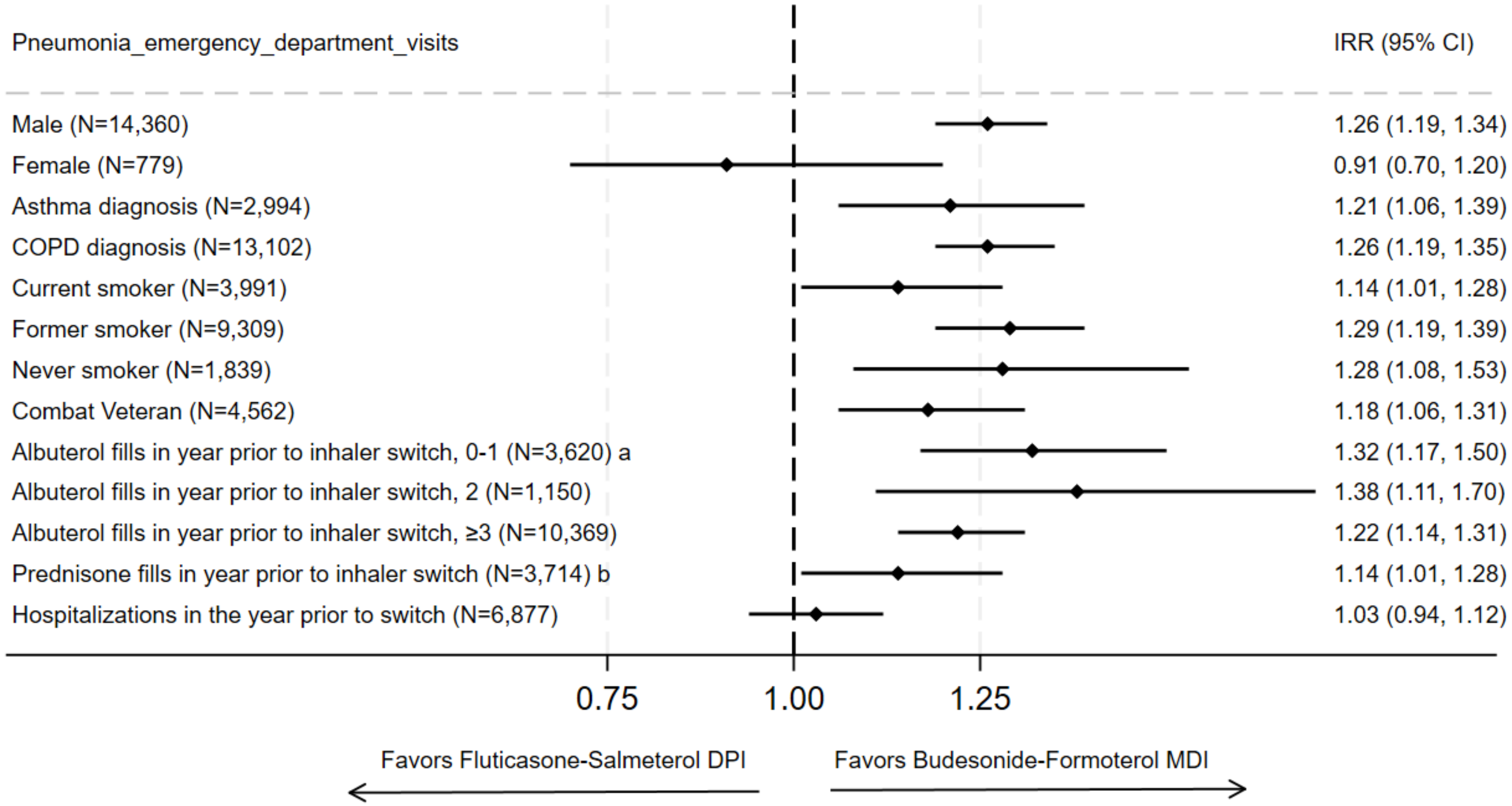

© 2025 Rabin AS et al. *JAMA Intern Med*.

Panel F

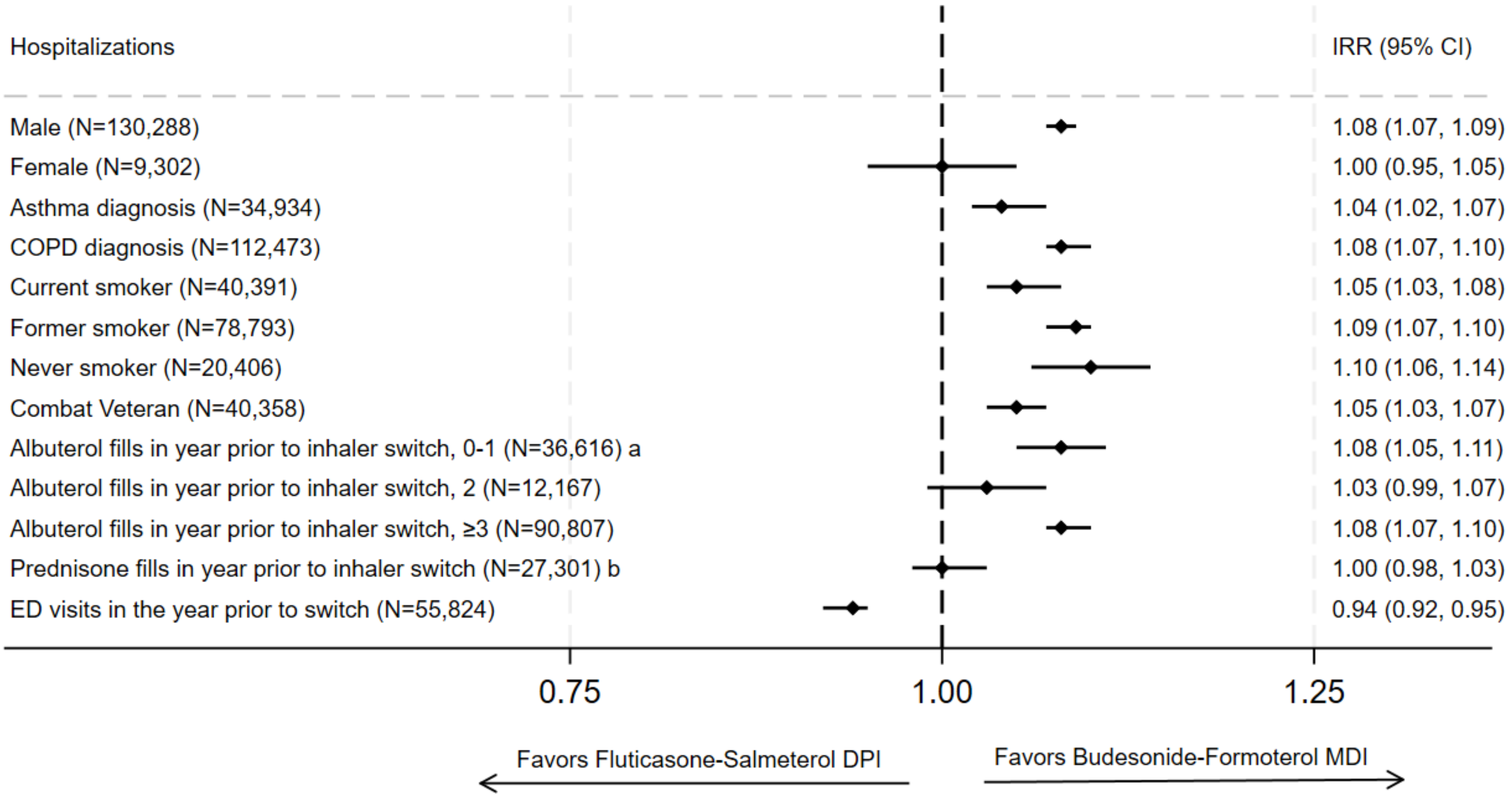

© 2025 Rabin AS et al. *JAMA Intern Med*.

Panel G

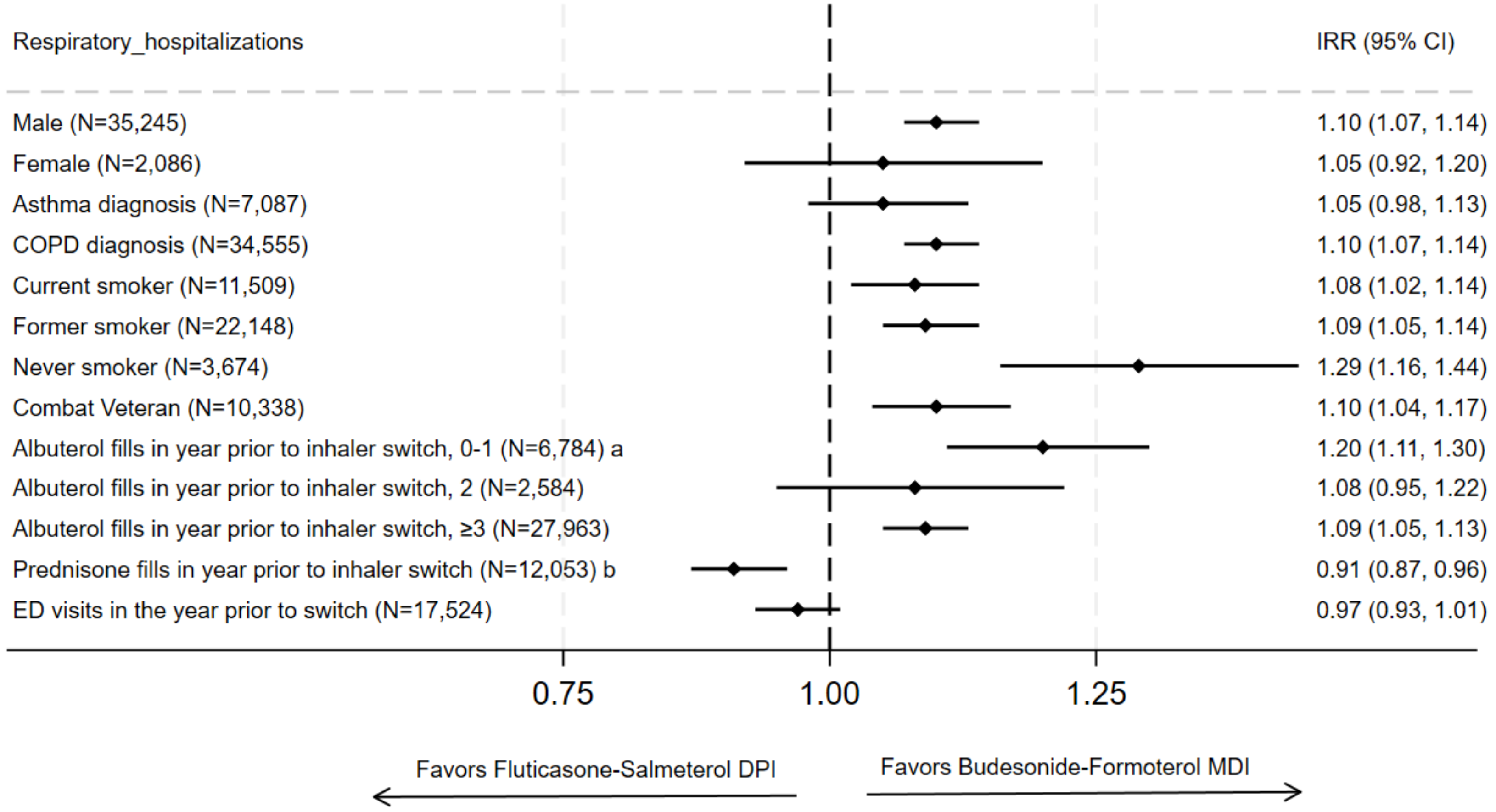

© 2025 Rabin AS et al. *JAMA Intern Med*.

Panel H

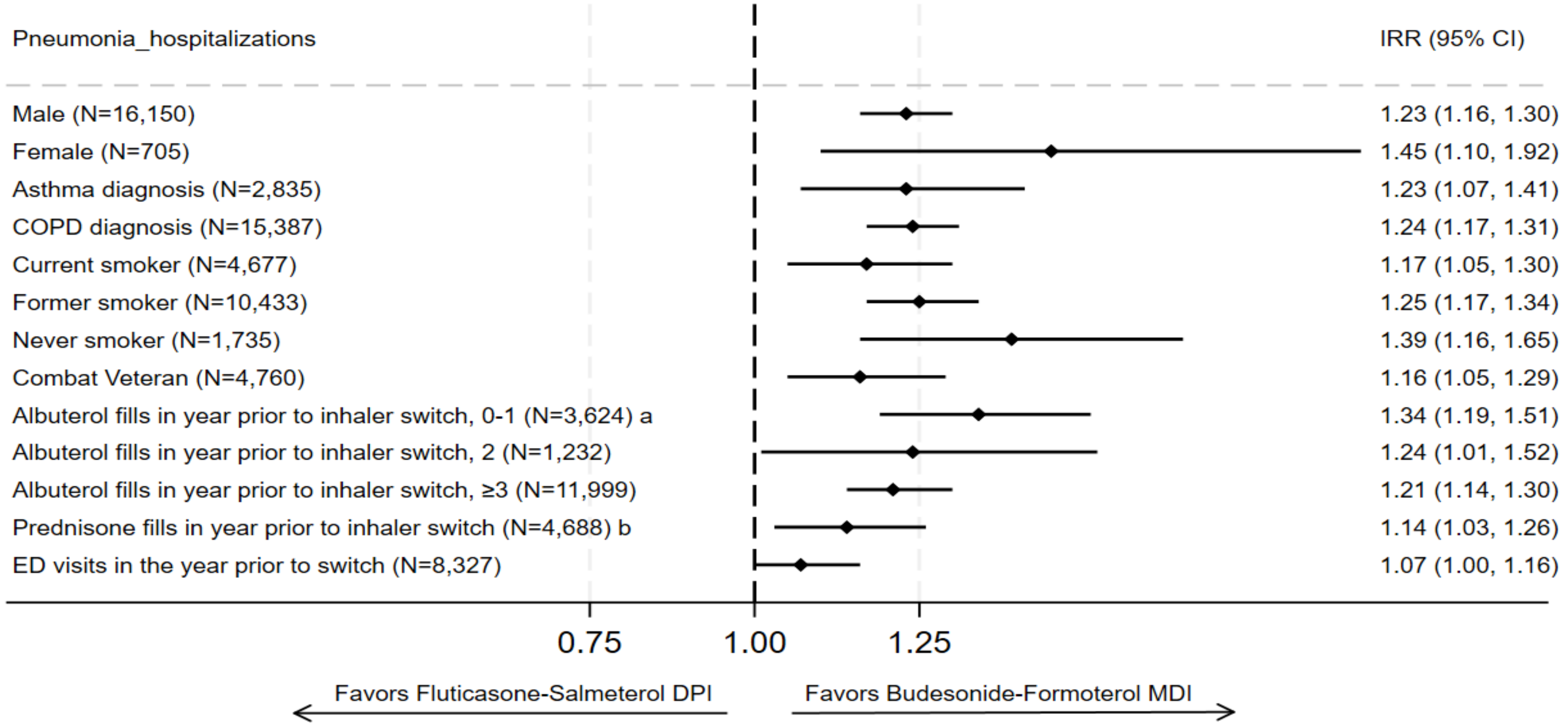

[a] Albuterol represented as inhaler-equivalent medication fills.

[b] Prednisone represented as discrete courses of the medication filled.

COPD indicates chronic obstructive pulmonary disease; DPI, dry-powder inhaler; IRR, incidence rate ratio; and MDI, metered-dose inhaler. Shown is the relative incidence of adverse health outcomes, stratified by subgroup, during periods of fluticasone–salmeterol use vs periods of budesonide–formoterol use among those who experienced the outcome of interest.

© 2025 Rabin AS et al. *JAMA Intern Med*.

eFigure 4. Self-Controlled Case Series Study Design, Expanded

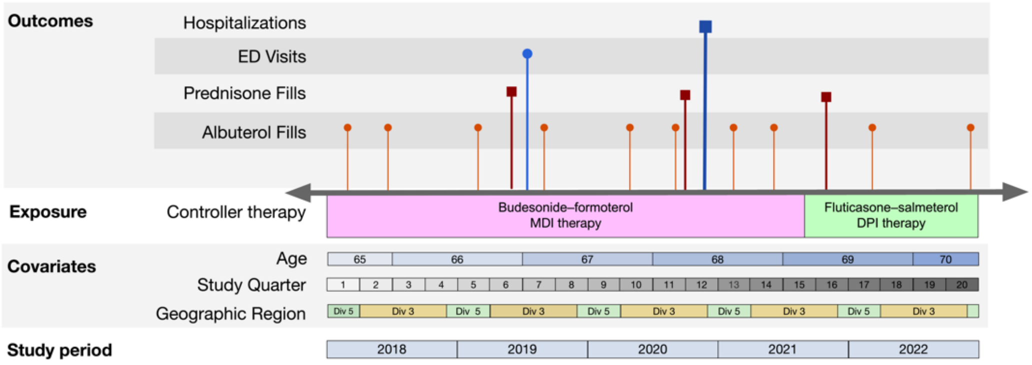

Patient i	ICS/LABA Group j	Age k	Study Quarter l	Census Division m	Interval Length e_{ijklm}	Number of Albuterol Fills n_{ijklm}	Number of Prednisone Fills o_{ijklm}	Number of ED visits p_{ijklm}	Number of Hosps. q_{ijklm}
1	1	65	1	5	90	1	0	0	0
1	1	65	2	3	90	1	0	0	0
1	1	66	3	3	90	0	0	0	0
1	1	66	4	3	60	0	0	0	0
1	1	66	4	5	30	0	0	0	0
1	1	66	5	5	90	1	0	0	0
1	1	66	6	3	90	0	1	0	0
1	1	67	7	3	90	1	0	1	0
1	1	67	8	3	60	0	0	0	0
1	1	67	8	5	90	0	0	0	0
1	1	67	9	5	90	0	0	0	0
1	1	67	10	3	90	1	0	0	0
1	1	68	11	3	90	1	1	0	0
1	1	68	12	3	60	0	0	0	1
1	1	68	12	5	30	0	0	0	0
1	1	68	13	5	90	1	0	0	0
1	1	68	14	3	90	1	0	0	0
1	2	69	15	3	45	0	0	0	0
1	2	69	15	3	45	0	0	0	0
1	2	69	16	3	60	0	1	0	0
1	2	69	16	5	30	0	0	0	0
1	2	66	17	5	90	1	0	0	0
1	2	66	18	3	90	0	0	0	0
1	2	66	19	3	90	0	0	0	0
1	2	66	20	3	60	0	0	0	0
1	2	66	20	5	30	1	0	0	0

ED indicates emergency department; Hosps, hospitalizations; and ICS/LABA, inhaled corticosteroid plus long-acting β-agonist. Shown is the timeline of exposures and outcomes for a hypothetical patient. The patient's observation time is split into smaller time intervals uniquely defined by exposure period and covariates, as demonstrated in the dataset for the hypothetical patient.

© 2025 Rabin AS et al. *JAMA Intern Med.*

eReferences.

1. Veterans Affairs Formulary Advisor. Accessed December 20, 2024. https://www.va.gov/formularyadvisor/class/RE109
2. Tirumalasetty J, Miller SA, Prescott HC, et al. Greenhouse gas emissions and costs of inhaler devices in the US. *JAMA*. Published online August 29, 2024. doi:10.1001/jama.2024.15331
3. Rabin AS, Weinstein JB, Whittington TN, et al. Implementation of a metered-dose inhaler to dry-powder inhaler national formulary transition. *JAMA Netw Open*. 2024;7(12):e2449234.
4. Rabin AS, Weinstein JB, Seelye SM, Whittington TN, Hogan CK, Prescott HC. Development and validation of a pulmonary function test data extraction tool for the US department of veterans affairs electronic health record. *BMC Res Notes*. 2024;17(1):115.
5. U.S. Environmental Protection Agency. Greenhouse gas equivalencies calculator. Published August 28, 2015. Accessed December 19, 2024. https://www.epa.gov/energy/greenhouse-gas-equivalencies-calculator

© 2025 Rabin AS et al. *JAMA Intern Med*.
