# Supplementary material for: Budesonide-Formoterol Metered-Dose Inhaler vs Fluticasone-Salmeterol Dry-Powder Inhaler
Source: JAMA Intern Med. 2025 Jul 7;185(8):1005–13. doi: 10.1001/jamainternmed.2025.2299 (PMC12235531; doi:10.1001/jamainternmed.2025.2299)
Supplement: Supplement 2. — Data Sharing Statement [file jamainternmed-e252299-s002.pdf]

Data Sharing Statement

Rabin. Budesonide-Formoterol Metered-Dose Inhaler vs Fluticasone-Salmeterol Dry-Powder Inhaler. *JAMA Intern Med*. Published July 07, 2025. doi:10.1001/jamainternmed.2025.2299

Data
Data available: No
